# Dual‐Layer Transcriptional‐Protein Regulation by HvAP2‐12 Represses HvAP2‐18 Activity to Fine‐Tune Barley Starch Synthesis

**DOI:** 10.1111/pbi.70338

**Published:** 2025-08-23

**Authors:** Jinjin Ding, Yulong Li, Jing Liu, Na Lin, David Seung, Qiang Xu, Yazhou Zhang, Huaping Tang, Pengfei Qi, Mei Deng, Jian Ma, Guoyue Chen, Jirui Wang, Yuming Wei, Qiantao Jiang

**Affiliations:** ^1^ State Key Laboratory of Crop Gene Exploration and Utilization in Southwest China, Triticeae Research Institute Sichuan Agricultural University Chengdu Sichuan China; ^2^ Faculty of Agriculture, Forestry and Food Engineering Yibin University Yibin Sichuan China; ^3^ John Innes Centre Norwich Research Park Norwich UK

**Keywords:** barley, *HvAP2‐12*, *HvAP2‐18*, starch synthesis, transcription factors

## Abstract

Starch content and composition are key factors determining yield and quality of barley, but the molecular mechanisms regulating barley starch synthesis remain unclear. In this study, we identified an APETALA2/Ethylene‐Responsive factor (AP2/ERF) family gene *HvAP2‐12*, which has typical transcription factor characteristics. Overexpression of *HvAP2‐12* had no effect in total starch and amylose content, whereas the gene‐edited mutant lines showed increased total starch and significantly decreased amylose content. Further analysis revealed that HvAP2‐12 does not directly bind to the promoter of major starch synthesis genes. However, it binds to another AP2/ERF family member, the *HvAP2‐18* promoter and interacts with the HvAP2‐18 protein. *HvAP2‐18* also affects starch synthesis in barley grains, and its overexpression increases total starch content while significantly reducing amylose content. *HvAP2‐18* binds directly to the promoters of key starch synthesis genes *HvAGP‐S1* and *HvSBE1*, activating their expression. Additionally, *HvAP2‐12* inhibits *HvAP2‐18* from binding to *HvAGP‐S1* and *HvSBE1* promoters. Based on these findings, we propose that *HvAP2‐12* and *HvAP2‐18* play critical roles in the regulation of starch genes and in determining barley grain quality.

## 1 Introduction

Barley ( *Hordeum vulgare* L.) is the world's fourth‐largest crop and is widely used in beer brewing and feed production. Starch is the major storage component of barley grains, constituting 50%–70% of the grain dry weight (Hu et al. 2021; Zhou 2009). Therefore, starch content directly influences grain weight, which is a critical component of yield. Additionally, starch composition and structure impact its physicochemical properties and hydrolysis characteristics, ultimately determining the nutritional value and processing quality of barley (Asare et al. 2011; Lockyer and Nugent 2017; Yu et al. 2017). Consequently, the content and characteristics of starch are critical determinants of barley yield and quality.

Starch is a macromolecule composed of polymerised α‐D‐glucose units. It is composed of amylopectin and amylose, which are polymers that differ in molecular structure (Junejo et al. 2022; Liu 2005). Amylopectin is the major polymer in starch that accounts for 65%–95% of total starch content, and is a highly branched polymer of α‐D‐glucose units linked by α‐1,4‐glucoside bonds in linear chains and α‐1,6‐glucoside bonds at branch points (Kowittaya and Lumdubwong 2014; Ma et al. 2007). In contrast, amylose is a linear polymer of α‐D‐glucose units linked by α‐1,4‐glucoside bonds and very few branches, and typically accounts for 5%–35% of total starch content (Jenkins and Donald 1995; Seung et al. 2015). Both amylopectin and amylose molecules are organised to form semi‐crystalline starch granules (Pérez and Bertoft 2010).

In cereal grains, starch synthesis begins with sucrose imported from photosynthetic tissues and is completed in the endosperm through a complex enzymatic pathway (Tetlow et al. 2015). ADP‐glucose pyrophosphorylase (AGPase) catalyses the production of ADP‐glucose (ADPG) from glucose‐1‐phosphate (G‐1‐P) in the cytoplasm. AGPase, a heterotetramer of large (AGP‐L) and small (AGP‐S) subunits, catalyses the first committed step of starch synthesis and is often considered the rate‐limiting enzyme in the process (Gu et al. 2021; Tiessen et al. 2002). ADPG is then transported to plastids, where amylose and amylopectin are formed (Thorbjørnsen et al. 1996). Granule‐bound starch synthase I (GBSSI) catalyses amylose synthesis within starch granules, while amylopectin synthesis occurs on the granule surface (Shapter et al. 2009). Soluble starch synthase (SS) isoenzymes (SSI‐III) elongate amylopectin chains of different lengths, and starch branching enzymes (SBEI and SBEII) create branch points by introducing α‐1,6‐glycosidic bonds. SBEI mainly forms long chains, while SBEII produces shorter, highly branched chains (Li et al. 2019; Regina et al. 2004).

Transcriptional regulation is essential for starch synthesis in the endosperm, but is currently poorly understood. In maize, the knockout mutants of *ZmNAC128* and *ZmNAC130*, which are direct regulators of six key starch synthesis genes, show reduced starch content (Chen et al. 2023). In rice, *RISBZ1* regulates genes controlling starch composition, while *OsMADS1* influences both starch content and the arrangement of starch granules within endosperms (Kawakatsu et al. 2009; Liu et al. 2023). *OsNAC25*, along with *OsNAC20* and *OsNAC26*, fine‐tunes starch synthesis by repressing starch‐related genes (Wang et al. 2024). In wheat, *TaNAC100* activates starch synthesis (Li et al. 2021), whereas *TaNAC019* suppresses it by targeting specific promoter elements (Gao et al. 2021). TaNF‐Y transcription factor complexes regulate starch synthesis indirectly by suppressing *TaNAC019*, thus promoting starch accumulation (Chen et al. 2024).

The AP2/ERF gene family, a large plant‐specific transcription factor family, plays significant roles in regulating plant growth and development (Ding et al. 2021). A few members of the AP2/ERF family have also been implicated in grain development and crop yield. For example, *OsERF115* acts as a transcriptional repressor, induced by ethylene, to regulate grain size and endosperm development in rice (Liu et al. 2022). In *Brachypodium distachyon* , the AP2/ERF transcription factor *BdDUO1* influences grain number per spike and reduces 1000‐grain weight (Wang et al. 2022). Rice Starch Regulator 1 (*RSR1*), another AP2/ERF transcription factor, negatively regulates starch synthesis gene expression (Fu and Xue 2010).

Despite these findings, studies on the role of AP2/ERF transcription factors in starch synthesis remain limited, and no research has yet explored their regulatory functions in barley. Further, little is known about functional interactions between different AP2/ERF members. In this study, building on prior analyses of the AP2/ERF family and transcriptome data, we identified *HvAP2‐12* as a candidate gene implicated in starch synthesis regulation. We show that it regulates starch accumulation by interacting with and regulating HvAP2‐18, which is the barley orthologue of RSR1 and a major regulator of starch synthesis genes. *HvAP2‐12* does not directly bind to promoters of starch synthesis genes but instead binds to *HvAP2‐18*, which binds to the promoters of starch synthesis genes (*HvAGP‐S*1 and *HvSBE1*) to regulate starch synthesis. *HvAP2‐12* inhibits the binding of HvAP2‐18 to these promoters, thereby modulating starch accumulation in barley grains.

## 2 Results

### 2.1 Identification of Candidate Transcription Factor *HvAP2‐12* for Barley Starch Regulation

**FIGURE 1:**
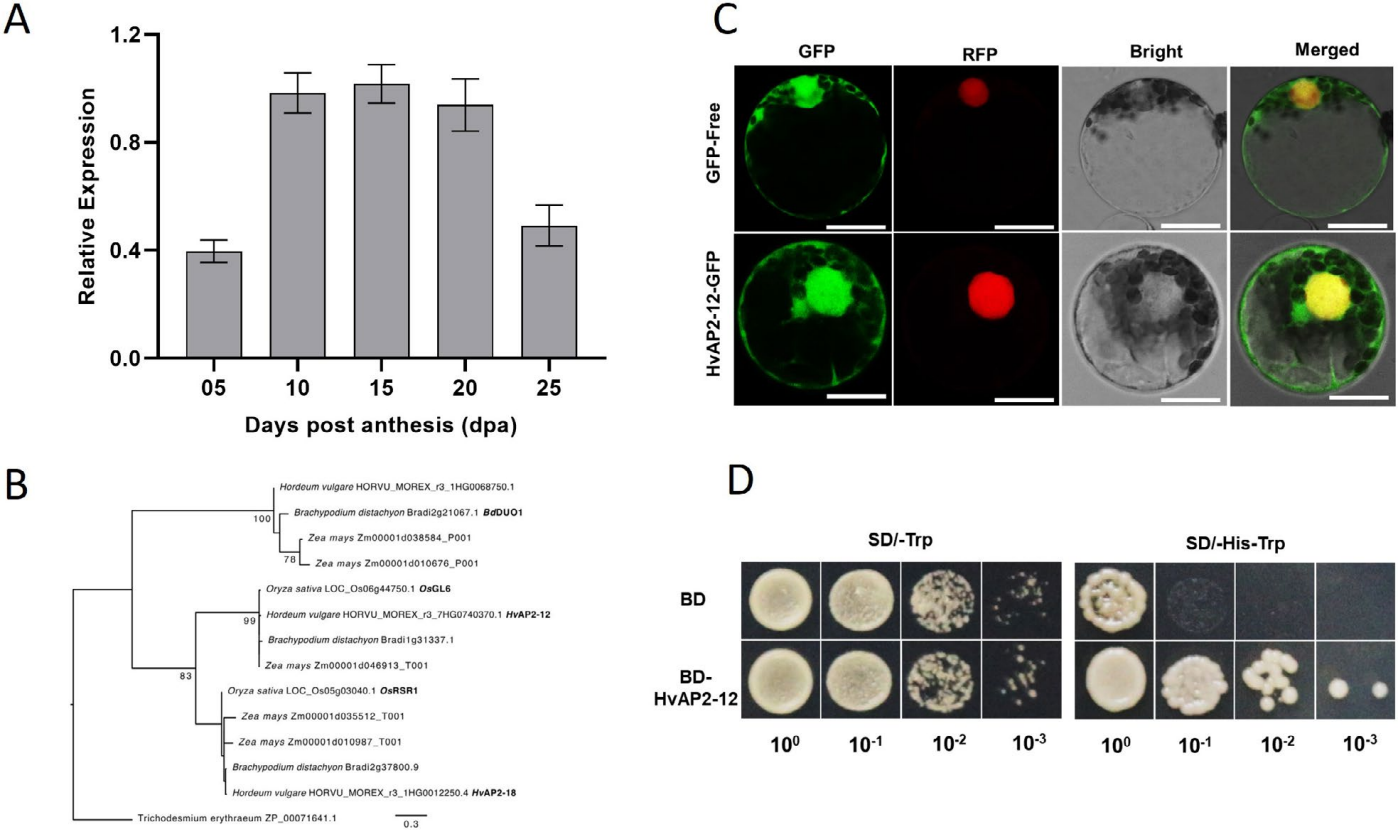
HvAP2‐12 Exhibits transcription factor characteristics. (A) Relative transcript levels of *HvAP2‐12* in the endosperm (from 05 to 25 DPA), as determined by RT‐qPCR. Data were normalised to barley β‐Actin and HvGAPDH, presented as mean ± SD (*n* = 3). (B) Homology comparison between HvAP2‐12 and homologous proteins from different species. The neighbour‐joining method (NJ) and a bootstrap value of 1000 were used to construct the phylogenetic tree. (C) Subcellular localisation of HvAP2‐12 in wheat protoplast cells. GFP: Green fluorescent protein. RFP was used as a nuclear marker. Scale bar: 20 μm. (D) HvAP2‐12 displays transcriptional activation activity. SD/−Trp: Tryptophan‐deficient medium; SD/−Trp‐His: Tryptophan histidineand—Deficient medium. BD: PGBKT7.

To identify transcription factors regulating barley starch synthesis, we previously performed a genome‐wide analysis of the AP2/ERF transcription factor family (Ding et al. 2021). Using published transcriptome data, we identified several AP2/ERF genes with high expression during grain development (Figure S1). The expression of *HvAP2‐12* is low during early grain development (5 days), peaks at the mid‐stage (10–20 days) and declines in the late stage (25 days) (Figure 1A).

The amino acid structure diagram shows that HvAP2‐12 contains two AP2/ERF domains (Figure S2). *HvAP2‐12*, the barley orthologue of rice *OsGL6*, promotes leaf trichome initiation in rice (Figure 1B). However, the role of *OsGL6* in the grain has not been explored (Xie et al. 2020). In addition, *HvAP2‐12* was found in a different clade to the other AP2/ERF family members that are reported to influence grain starch and size in other cereals: rice *RSR1* (the orthologue of barley *HvAP2‐18*) and *Brachypodium distachyon Duo* (Fu and Xue 2010; Wang et al. 2022). This suggests that *HvAP2‐12* is an AP2/ERF family member that has not been implicated in grain starch synthesis (Figure 1B).

The subcellular localisation of HvAP2‐12 was analysed by transiently expressing a fusion protein construct of HvAP2‐12 and green fluorescent protein (GFP) in *Nicotiana benthamiana* leaves and wheat protoplasts. GFP fluorescence signals were observed in the nucleus and cytoplasm of both *N. benthamiana* leaves and wheat protoplasts (Figures 1C, S3), indicating that HvAP2‐12 localises to the nucleus and cytoplasm.

HvAP2‐12 transactivation activity was assessed by transforming a fusion construct of HvAP2‐12 and the GAL4 transcription factor DNA‐binding domain (BD) into yeast. Yeast growth and selection signals on the selective medium confirmed that HvAP2‐12 exhibits transactivation activity (Figure 1D).

### 2.2 *HvAP2‐12* Regulates Starch and Amylose Content in Grains

To investigate the function of *HvAP2‐12* in barley, we created CRISPR/Cas9 knockouts (*hvap2‐12*) and native promoter‐driven overexpression lines (HvAP2‐12) in cv. Golden Promise. A conserved sgRNA targeting exon 2 produced one same frameshift mutant line with premature gene termination (Figure S4). The overexpression construct, validated by GUS fusion in tobacco (Figure S5), yielded three transgenic lines with 0.84‐, 3.61‐ and 2.74‐fold *HvAP2‐12* expression versus WT (Figure S6).

**FIGURE 2:**
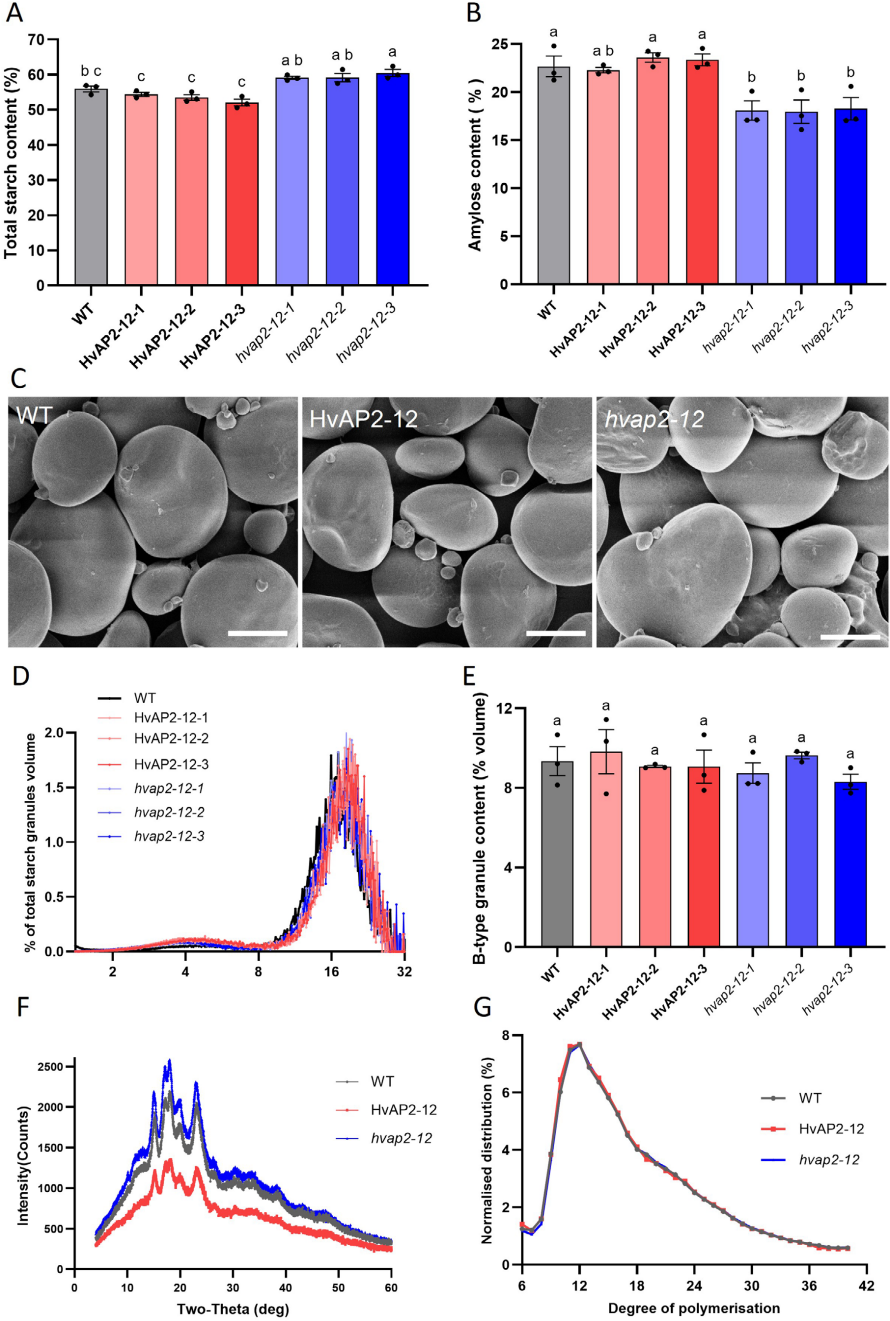
Analysis of the physicochemical properties of grain starch in *HvAP2‐12* overexpression and mutant lines. (A) Total starch content in the overexpression, mutant and WT lines. (B) Amylose content in the overexpression, mutant and WT lines. (C) Scanning electron microscopy (SEM) of starch granules in the mature grain endosperm of *HvAP2‐12* overexpression, mutant and WT lines. Scale bar: 10 μm. (D) Granule size distributions were determined using a Coulter counter, with data expressed as relative % volume (of total starch) versus. granule diameter. (E) B‐type granule volume (% of total starch) was extracted from the relative volume versus. diameter plots by fitting a bimodal mixed normal distribution. (F) X‐ray diffraction (XRD) profiles of starch, HvAP2‐12‐2 and *hvap2‐12‐2* lines were selected for the determination. (G) Distribution of amylopectin chain length in HvAP2‐12, the selected lines are the same as in D).

No significant differences were observed in plant/grain morphology, hundred‐grain weight or grain dimensions (length/width) between WT and transgenic lines (Figure S7A–H), indicating *HvAP2‐12* does not regulate these traits. The total starch content decreased in all three overexpression lines but increased in the gene‐edited lines (Figure 2A). No significant change in amylose content was observed in the *HvAP2‐12* overexpression lines (HvAP2‐12‐1, HvAP2‐12‐2 and HvAP2‐12‐3) relative to wild‐type controls (Figure 2B). Conversely, linear amylose content decreased very significantly in all mutants (Figure 2B). These results indicate that *HvAP2‐12* significantly influences starch content and the amylose content in barley grains.

### 2.3 *HvAP2‐12* Does Not Affect Any Starch Properties

Since altered *HvAP2‐12* expression changed total starch and amylose levels, we examined starch granule morphology. SEM analysis showed comparable granule structures in overexpression/mutant lines and wild‐type (Figure 2C). Coulter counter analysis revealed similar size distributions across all lines, with distinct A/B‐type granule peaks and no significant differences in B‐type content (Figure 2D,E). These results indicate *HvAP2‐12* does not influence starch granule morphology.

XRD analysis of *HvAP2‐12* transgenic lines (overexpression, mutant, WT) displayed characteristic A‐type crystallinity peaks at 15°, 23° 2θ and unresolved 17°–18° doublets, consistent with normal cereal starch patterns (Figure 2F). However, quantitative assessment demonstrated that overexpression lines exhibited significantly lower starch crystallinity than WT; whereas the *hvap2‐12* mutant showed increased crystallinity relative to WT (Figure S8). This establishes that *HvAP2‐12* specifically modulates starch crystallinity without altering crystalline type. As *HvAP2‐12* affects the amylose content, we further analysed the properties of the starch polymers. We first tested whether the chain length distribution structure of amylopectin was altered in the mutant and overexpression lines using High‐Performance Anion‐Exchange Chromatography (HPAEC) following normalisation of data obtained from overexpressed lines, mutant lines and wild‐type (WT) controls spanning stages DP6 to DP42, no distinct variations in chain length distribution patterns emerged among the different genetic variants (Figure 2G). HvAP2‐12‐2, hvap2‐12‐2 and WT lines were not significantly different.

### 2.4 *HvAP2‐12* Affects the Expression of Multiple Starch Synthase Genes

**FIGURE 3:**
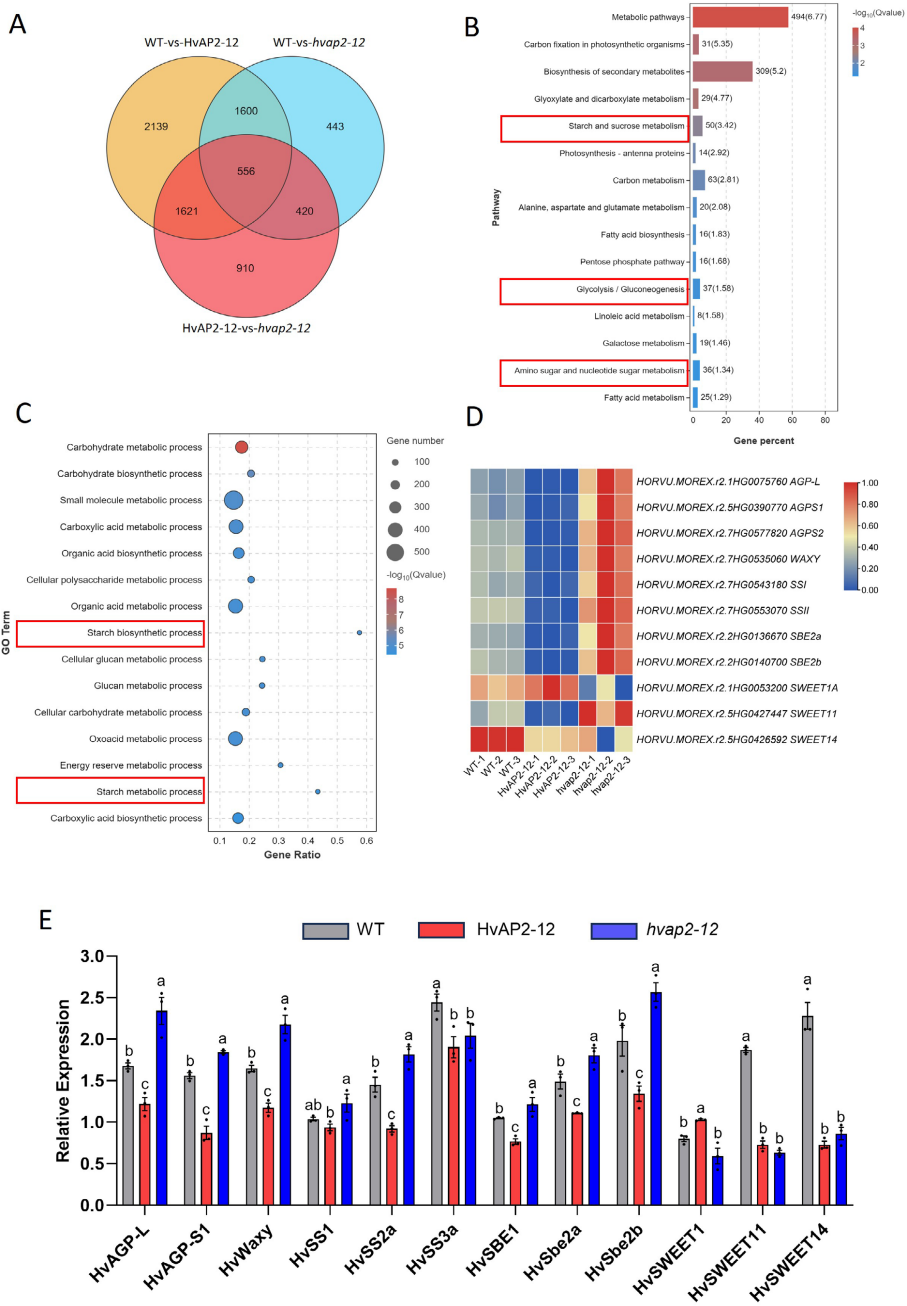
*HvAP2‐12* affects the expression levels of starch and sucrose synthesis genes. (A) Venn diagram showing the number of DEGs in overexpression, mutant and WT lines. Set up three comparison groups: WT versus HvAP2‐12, WT versus *hvap2‐12*, HvAP2‐12 versus *hvap2‐12*. The overlapping gene set is statistically significant (Fisher's exact test: *P* < 0.0001, OR = 17.42), calculated against 34 919 background expressed genes. (B, C) KEGG pathways and GO enrichment of *HvAP2‐12* overexpression, mutant and WT lines. The pathways associated with starch and sucrose synthesis are circled in the red box. (D) Heatmap showing the expression levels of starch synthesis and starch grain‐related genes in *HvAP2‐12* overexpression, knockout and WT lines. (E) Relative expression levels of *HvAGP‐L*, *HvAGP‐S1*, *HvSS1*, *HvSS2a*, *HvSS3a*, *HvSBE1*, *HvSBE2a*, *HvSBE2b*, *HvSWEET1*, *HvSWEET11* and *HvSWEET14* in *HvAP2‐12* overexpression and mutant lines compared to WT, estimated by RT‐qPCR and normalised to barley *β‐actin* and *HvGAPDH*. Data are presented as mean ± SD from three biological replicates. Values with different letters indicate significant differences, as determined by one‐way ANOVA with Tukey's post hoc test (*p* < 0.05).

To investigate *HvAP2‐12*'s role in starch metabolism, we conducted RNA‐seq on 15 dpa seeds from three overexpression (HvAP2‐12‐1/‐2/‐3) and three mutant lines. PCA showed genotype‐specific clustering of replicates (WT, HvAP2‐12, *hvap2‐12*) (Figure S9A). Comparisons revealed 5916 (WT vs. HvAP2‐12), 3019 (WT vs. *hvap2‐12*) and 3507 (HvAP2‐12 vs. *hvap2‐12*) DEGs (|log_2_FC| > 1, FDR < 0.05). The HvAP2‐12 versus *hvap2‐12* comparison identified 1601 upregulated and 1906 downregulated genes (Figures S9B and 3A), with 556 DEGs shared across all comparisons (Figure 3A). KEGG/GO analyses of DEGs highlighted enrichment in metabolic pathways, particularly starch/sucrose metabolism (50 DEGs) and starch biosynthesis (25 total DEGs) (Figure 3B,C). This implicates *HvAP2‐12* in starch synthesis regulation.

Key starch‐related genes exhibited expression‐phenotype correlations: AGPase subunits (*AGP‐L*, *AGPS1/2*), *Waxy*, *SS1/2* and *SBE2a/b* were downregulated in overexpression lines and upregulated in mutants, aligning with their starch content changes (Figure 3D). *HvSWEET11* and *HvSWEET14* both exhibited concordant downregulation in the overexpression lines and the knockout mutants, whereas *HvSWEET1A* was upregulated in overexpression lines and downregulated in mutants (Figure 3E). Notably, *GW2* (grain size) and *FLO6* (granule morphology) displayed inverse expression trends despite unchanged phenotypes. RT‐qPCR validation in 15 dpa endosperms (HvAP2‐12‐2 overexpression and *hvap2‐12‐2* mutant lines) confirmed starch synthesis gene expression trends: *HvAGP‐L/S1*, *HvWaxy*, *HvSS1/2a*, *HvSBE1/2a/2b* were downregulated in overexpression but upregulated in mutants. *HvSS3a* showed consistent downregulation across both transgenic genotypes (Figure 3E).

Dual‐Luciferase assays testing *HvAGP‐L/S*, *HvWaxy*, *HvSS2a* and *HvSBE2b* promoters showed no significant LUC/REN ratio changes versus controls, indicating HvAP2‐12 does not directly regulate these genes (Figure S10).

### 2.5 HvAP2‐12 Inhibits *HvAP2‐18* and Interacts With HvAP2‐18 Protein

**FIGURE 4:**
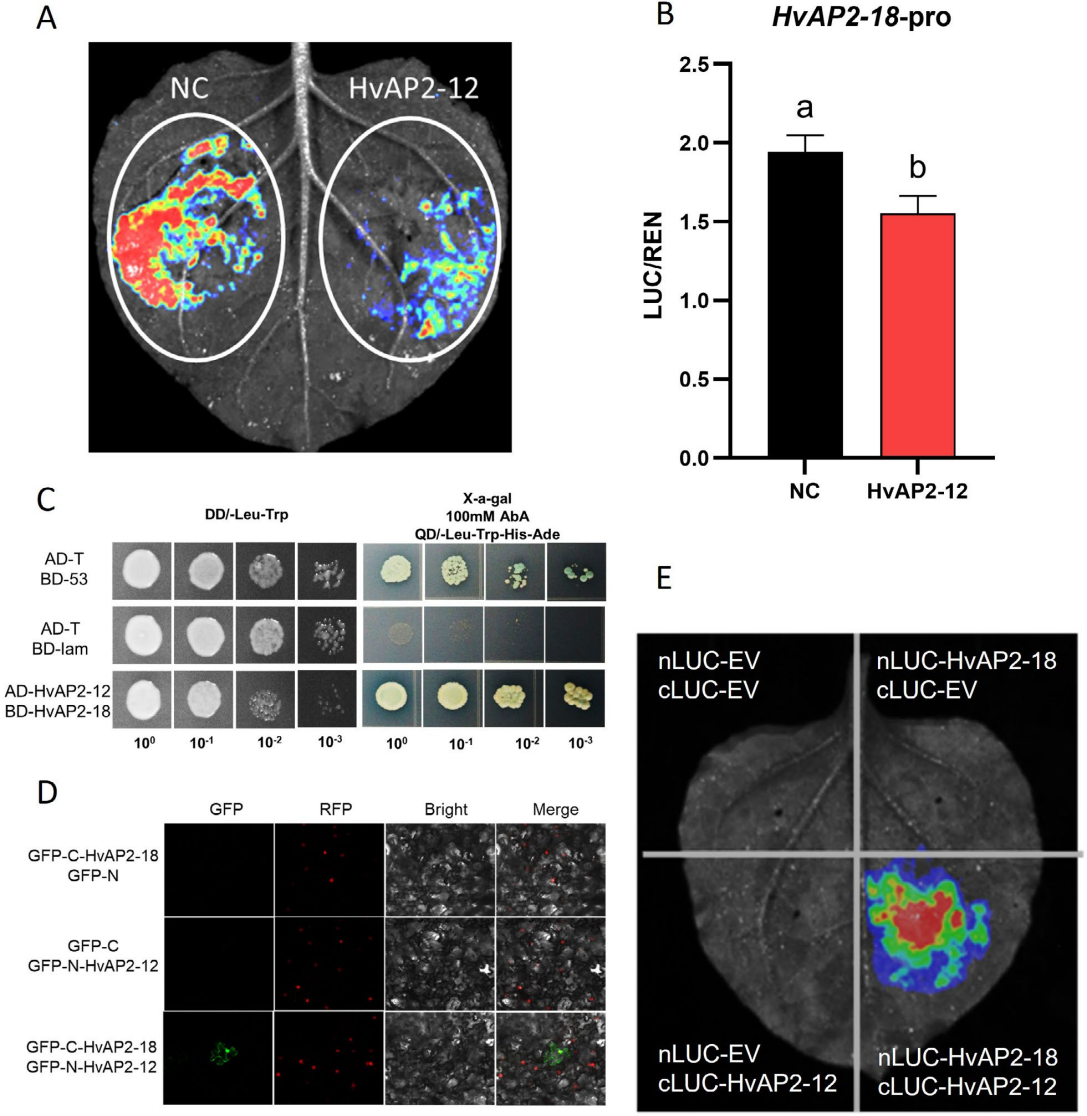
HvAP2‐12 inhibits the expression of *HvAP2‐18*. (A, B) Dual‐luciferase transcriptional activity assays to assess the effect of HvAP2‐12 on *HvAP2‐18* expression. The LUC/REN ratio on the y axis indicates the signal detected for firefly luciferase (LUC) and *Renilla reniformis* luciferase (REN) activity. Error bars represent standard deviation (SD) for three replicates. Values with different letters indicate significant differences, as determined by one‐way ANOVA with Tukey's post hoc test (*p* < 0.05). (C) Detection of HvAP2‐12 and HvAP2‐18 protein interaction by the yeast two‐hybrid system. Cotransformation with T‐antigen and Lam served as a negative control (CK‐), while cotransformation with T‐antigen and p53 was used as a positive control (CK+). (D) BiFC assay showing the interaction between HvAP2‐12 and HvAP2‐18 in Nicotiana benthamiana leaves. RFP was used as a nuclear marker. (E) The LUC assay measured the interaction intensity between HvAP2‐12 and HvAP2‐18.

Given that *HvAP2‐12* did not directly bind the promoters of starch synthesis genes, we hypothesized that it could indirectly regulate these genes by regulating other transcription factors. Therefore, we comprehensively screened differentially expressed genes (DEGs) in the *HvAP2‐12* transgenic transcriptome dataset for barley homologues of known starch metabolism regulators, including members of the AP2/ERF, NAC, MADS and bZIP families. Six candidate transcription factors were selected for validation (Figure S11), and their differential expression was confirmed via qRT‐PCR (Figure S12). To assess the interaction between *HvAP2‐12* and these six candidate genes, dual‐luciferase reporter (DLR) assays were performed using constructs containing the upstream promoter regions (1500 bp upstream of the ATG start codon) of each candidate gene. Among these, only *HvAP2‐18* showed a significant response: the LUC/REN ratio was significantly reduced compared to the Negative Control (Figures 4A,B and S13), suggesting *HvAP2‐18* is directly regulated by *HvAP2‐12*.

**FIGURE 5:**
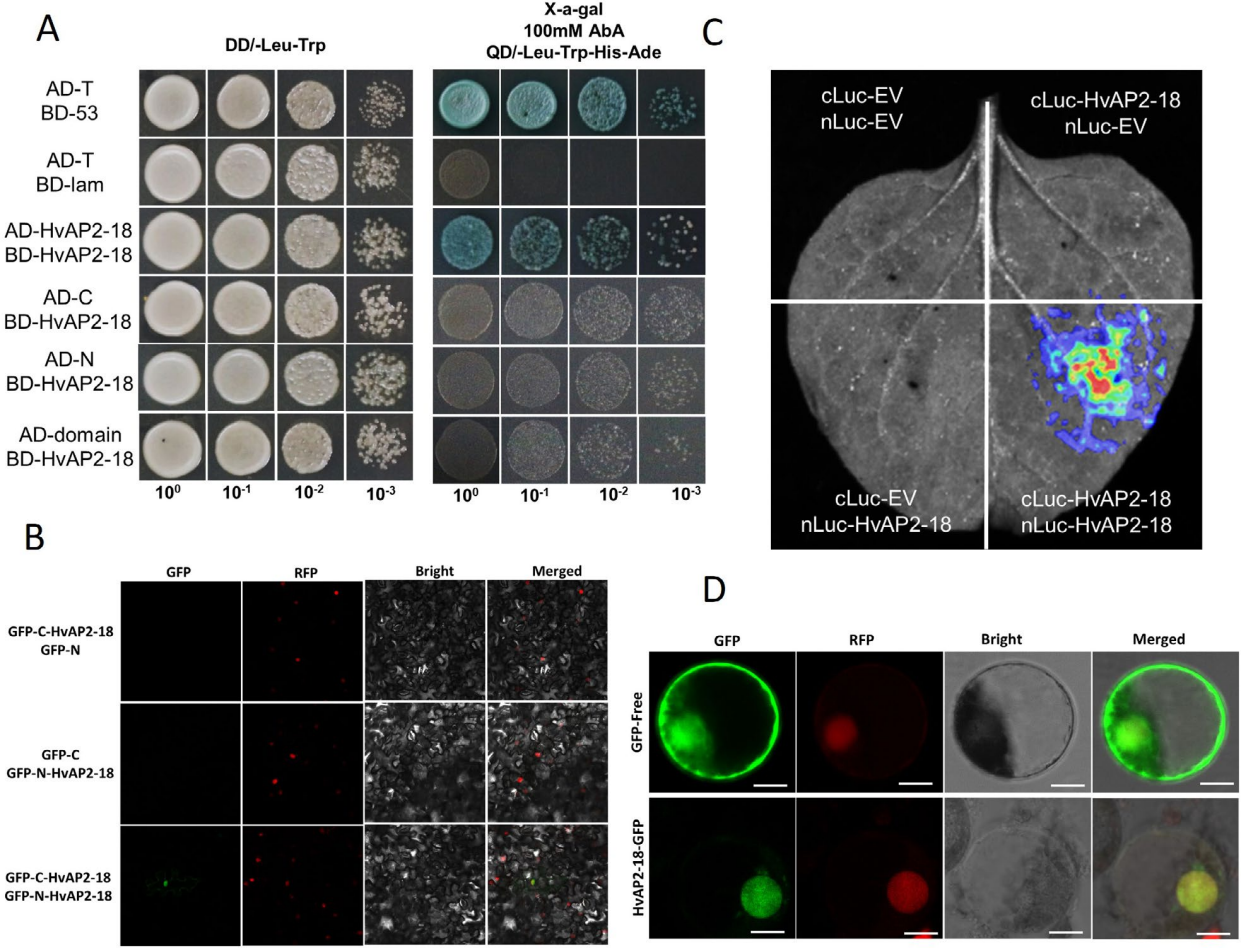
HvAP2‐18 is localised to the nucleus and exists as a dimer. (A) Detection of the HvAP2‐18 protein homodimer by the yeast two‐hybrid system. Cotransformation with T‐antigen and p53 was used as a positive control (CK+), while cotransformation with T‐antigen and Lam served as a negative control (CK‐). SD/−Leu‐Trp: Leu, tryptophan‐deficient medium; SD/−Leu‐Trp‐His‐Ade + X‐α‐gal: Leu, tryptophan, histidine and alanine‐deficient medium with α‐galactosidase. (B) BiFC assay showing the HvAP2‐18 protein homodimer in Nicotiana benthamiana leaves. RFP was used as a nuclear marker. (C) The LUC assay confirms the HvAP2‐18 protein homodimer. (D) Subcellular localisation of HvAP2‐18 in wheat protoplast cells. GFP: Green fluorescent protein. RFP was used as a nuclear marker. Scale bar: 20 μm.

We conducted a yeast two‐hybrid assay, which showed that AD‐HvAP2‐12 and BD‐HvAP2‐18 formed blue colonies on QD/−Leu‐Trp‐His‐Ade + X‐α‐gal medium, suggesting that the two proteins interact (Figure 4C). To confirm the interaction in planta, BiFC assays showed fluorescence when GFP‐N‐HvAP2‐12 and GFP‐C‐HvAP2‐18 were co‐expressed in *Nicotiana benthamiana* leaves, but not in the controls (Figure 4D). Similarly, we used a split Luciferase assay, where luminescence was detected when nLUC‐HvAP2‐12 and cLUC‐HvAP2‐18 were co‐expressed in *Nicotiana benthamiana* leaves, but not in any of the controls (Figure 4E). Taken together, these experiments confirm a protein–protein interaction between HvAP2‐12 and HvAP2‐18. In addition to forming heterodimers, we also demonstrated using yeast two‐hybrid, LUC and BiFC assays that HvAP2‐18 forms homodimers (Figure 5A–C).

Subcellular localisation studies using GFP‐tagged HvAP2‐18 protein expressed in tobacco and wheat protoplasts showed that it locates to the nucleus in both species (Figure 5D and Figure S14).

### 2.6 *HvAP2‐18* Regulates Grain Size

We used qPCR to detect the expression of *HvAP2‐18* in the T2 generation of the *HvAP2‐18* overexpression lines. Three overexpression lines (HvAP2‐18‐4, HvAP2‐18‐8 and HvAP2‐18‐10) had 1.94, 4.26 and 10.16 times higher *HvAP2‐18* expression than the WT, respectively (Figure S15). For gene editing to produce knockout mutants, a target site was designed in the fourth exon of *HvAP2‐18*, resulting in three different mutations that all led to early termination of the protein (Figure S16).

**FIGURE 6:**
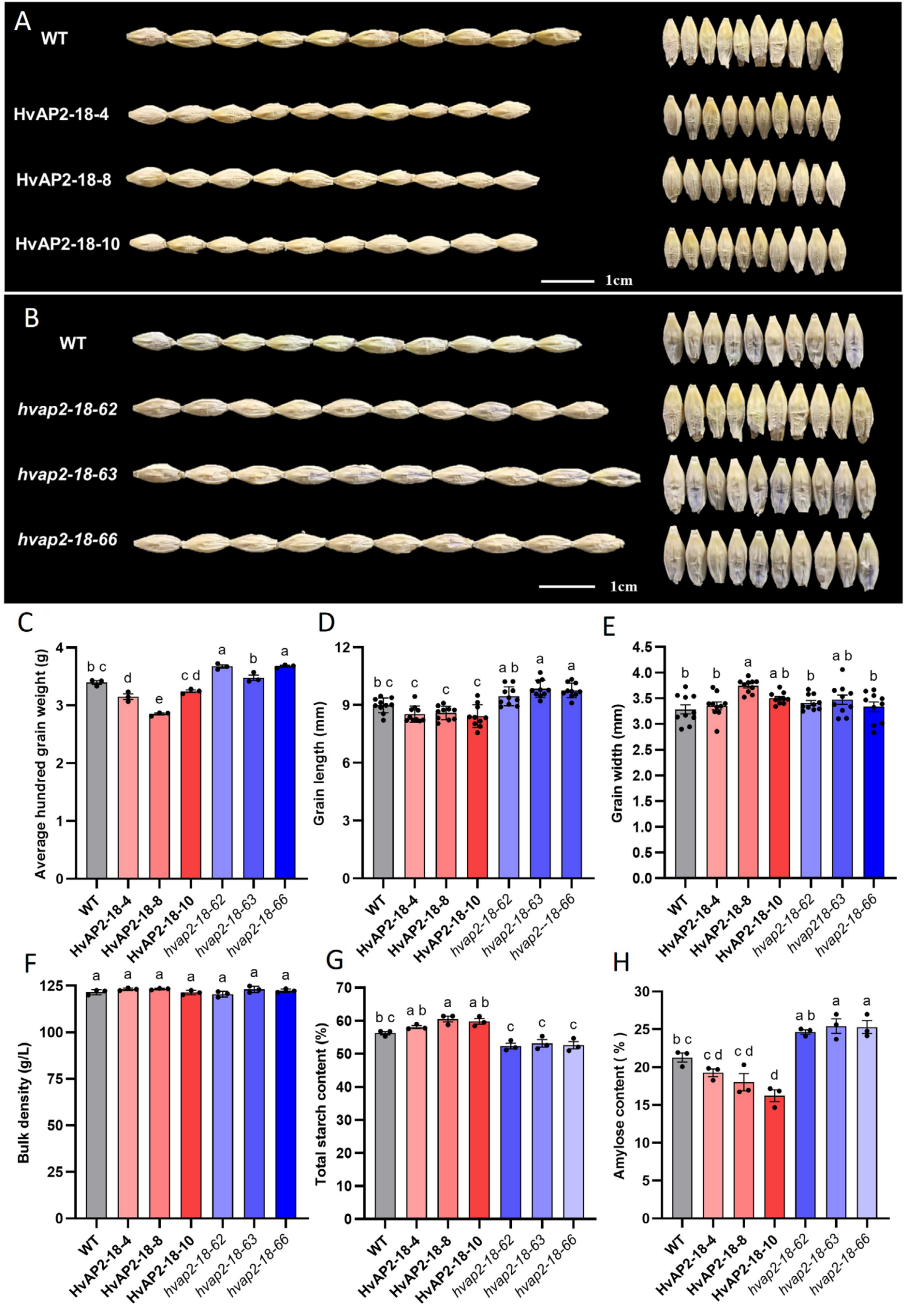
Grain morphology and starch content in *HvAP2‐18* overexpression and mutant lines. (A, B) Morphological observations of plants and grains from *HvAP2‐18* overexpression, mutant and WT lines. Average hundred‐grain weight (C), grain length (D), width (E) and bulk density (F) of overexpression, mutant and WT lines. (G) Total starch content in the overexpression, mutant and WT lines. (H) Amylose content in the overexpression, mutant and WT lines. Data are presented as mean ± SD from three biological replicates. Values with different letters indicate significant differences, as determined by one‐way ANOVA with Tukey's post hoc test (*p* < 0.05).

Like the *HvAP2‐12* lines, there were no significant differences in plant morphology between the *HvAP2‐18* overexpression and mutant lines at either tillering or maturity (Figure S17). However, unlike the *HvAP2‐12* lines, grain morphology varied significantly among the *HvAP2‐18* lines (Figure 6A,B). The 100‐grain weight increased significantly in all three mutant lines but decreased in most overexpression lines, except for the line with the lowest overexpression (HvAP2‐18‐4) (Figure 6C). The grain length of overexpression lines was significantly reduced, whereas the mutant lines exhibited a significant increase in grain length compared to WT (Figure 6D). No consistent significant changes in grain width were observed among all overexpression or all mutant lines (Figure 6E). Unit weight showed no significant differences across the lines (Figure 6F). These results indicate that *HvAP2‐18* affects grain length and 100‐grain weight without altering overall plant morphology.

### 2.7 *HvAP2‐18* Affects Starch Content and Composition but Not Starch Structure

Most of the overexpression line for *HvAP2‐18* had increased total starch compared to WT (except for the HvAP2‐18‐8 line significantly increased), while all three mutants had decreased starch content (Figure 6G). There was no consistent change in amylose content following *HvAP2‐18* overexpression, as it was significantly lower than WT in HvAP2‐18‐10, but not significantly changed in HvAP2‐18‐4 and HvAP2‐18‐8. In contrast, amylose content was significantly increased in *hvap2‐18‐63* and *hvap2‐18‐66* mutant lines (Figure 6H). These findings suggest that *HvAP2‐18* affects both starch synthesis and its composition, but in an opposite manner to *HvAP2‐12*.

The mutant or overexpression of *HvAP2‐18* did not alter starch granule morphology when viewed under SEM (Figure S18A); nor did it affect granule size distributions when quantified with a Coulter counter (Figure S18B,C). XRD analysis also showed no alterations to granule crystalline arrangement, with the typical A‐type allomorph observed with strong reflections at approximately 15° and 23°2θ and unresolved peaks at 17° and 18°2θ in all lines, consistent with *HvAP2‐12* properties (Figure S19A). The chain length structure of the *HvAP2‐18* lines showed no change. Measurement of amylopectin chain length distribution via HPAEC revealed no significant differences between *HvAP2‐18* overexpression/mutant lines and wild‐type controls (Figure S19B).

### 2.8 Binding of HvAP2‐18 to the Promoters of Starch Synthesis Genes

**FIGURE 7:**
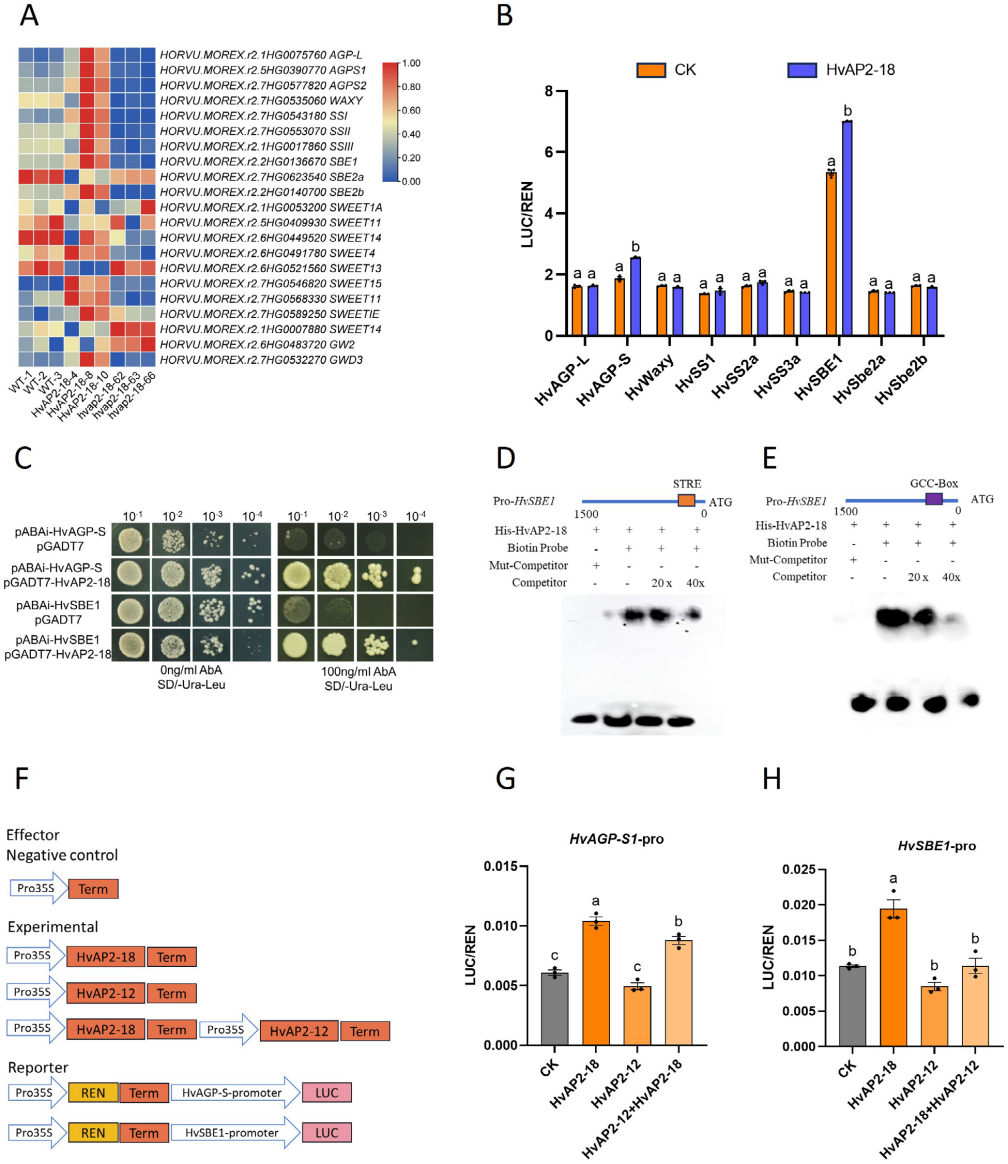
HvAP2‐12 inhibits the binding of HvAP2‐18 to starch synthase gene promoters *HvAGP‐S1* and *HvSBE1*. (A) Heatmap of differential genes involved in starch and sucrose synthesis. (B) Dual‐luciferase reporter (DLR) validation of HvAP2‐18 binding to differential gene promoters in starch synthesis. (C) Yeast one‐hybrid assay validating that HvAP2‐18 binds to starch synthase gene promoters *HvAGP‐S1* and *HvSBE1*. (D, E) EMSA verified the binding of HvAP2‐18 to the GCC‐box and STRE in the promoters of *HvSBE1*. Orange box highlights predicted cis‐elements STRE and the purple box highlights predicted cis‐elements GCC‐box. (F–H) The transient DLR assay of HvAP2‐12 inhibits the binding of HvAP2‐18 to the promoter *HvAGP‐S1* and *HvSBE1*. Data are presented as mean ± SD from three biological replicates. Values with different letters indicate significant differences, as determined by one‐way ANOVA with Tukey's post hoc test (*p* < 0.05).

Transcriptomic profiling of 15 DPA grains from overexpression (OE), mutant and wild‐type (WT) lines identified downstream targets of HvAP2‐18. Comparisons of WT versus HvAP2‐18, WT versus *hvap2‐18* and HvAP2‐18 versus *hvap2‐18* revealed 4840, 4063 and 6494 differentially expressed genes (DEGs), respectively, with 2343 upregulated and 4151 downregulated genes in the HvAP2‐18 versus *hvap2‐18* group (Figure S20A,B). A total of 1080 DEGs overlapped across comparisons. KEGG/GO enrichment highlighted DEGs in plant growth/development pathways, including 52 starch/sucrose synthesis and 49 glycolysis/metabolism genes (Figure S20C,D). Heatmap analysis showed upregulation of nine starch synthesis genes in OE lines and their downregulation in mutants (Figure 7A), overlapping with DEGs from *HvAP2‐12* perturbations. The atypical behaviour of the HvAP2‐18‐4 line (likely due to low overexpression) prompted validation using qRT‐PCR in HvAP2‐18‐8 and *hvap2‐18‐63* lines (Figure S21), confirming *HvAP2‐18*'s role in starch synthesis regulation.

Dual‐luciferase reporter (DLR) assays demonstrated HvAP2‐18 binding to *HvAGP‐S1* and *HvSBE1* promoters, enhancing their expression (Figure 7B). Promoter truncation (3 × 500 bp segments) revealed preferential binding to the first segment of *HvAGP‐S1* and the third segment of *HvSBE1* (Figure S22), further validated by yeast one‐hybrid assays (Figure 7C). Analysis of the promoter elements of *HvAGP‐S1* and *HvSBE1* revealed that both the first segment of the *HvAGP‐S1* promoter and the third segment of the *HvSBE1* promoter contain STRE elements and GCC‐box elements (binding sites for the AP2/ERF family) (Figure S23). We analysed the binding elements in these two promoter regions and found that they both contained the GCC‐box and STRE of the AP2/ERF family. Therefore, we used EMSA to verify that HvAP2‐18 could specifically bind to the GCC‐box and STRE in the promoters of *HvSBE1*, respectively (Figure 7D,E). While HvAP2‐12 does not directly bind these promoters, it significantly reduced the LUC/REN ratio of *HvAP2‐18*‐promoter interactions (Figure 7F–H), indicating suppression of HvAP2‐18's regulatory activity.

### 2.9 HvAP2‐12 Represses HvAP2‐18 to Orchestrate Starch Modulation

Double overexpression (OE: HvAP2‐12‐2/HvAP2‐18‐8) and mutant (*hvap2‐12‐2/hvap2‐18‐63*) lines were generated to study HvAP2‐12/HvAP2‐18 interactions. The expression levels of *HvAP2‐12* and *HvAP2‐18* in each overexpression line were detected by qRT‐PCR (Figure S24). Double OE lines showed no significant changes in grain weight, length or width versus WT; whereas double mutants exhibited increased 100‐grain weight and grain length (no width alteration), mirroring *HvAP2‐18* single mutants (Figure S25A–C).

**FIGURE 8:**
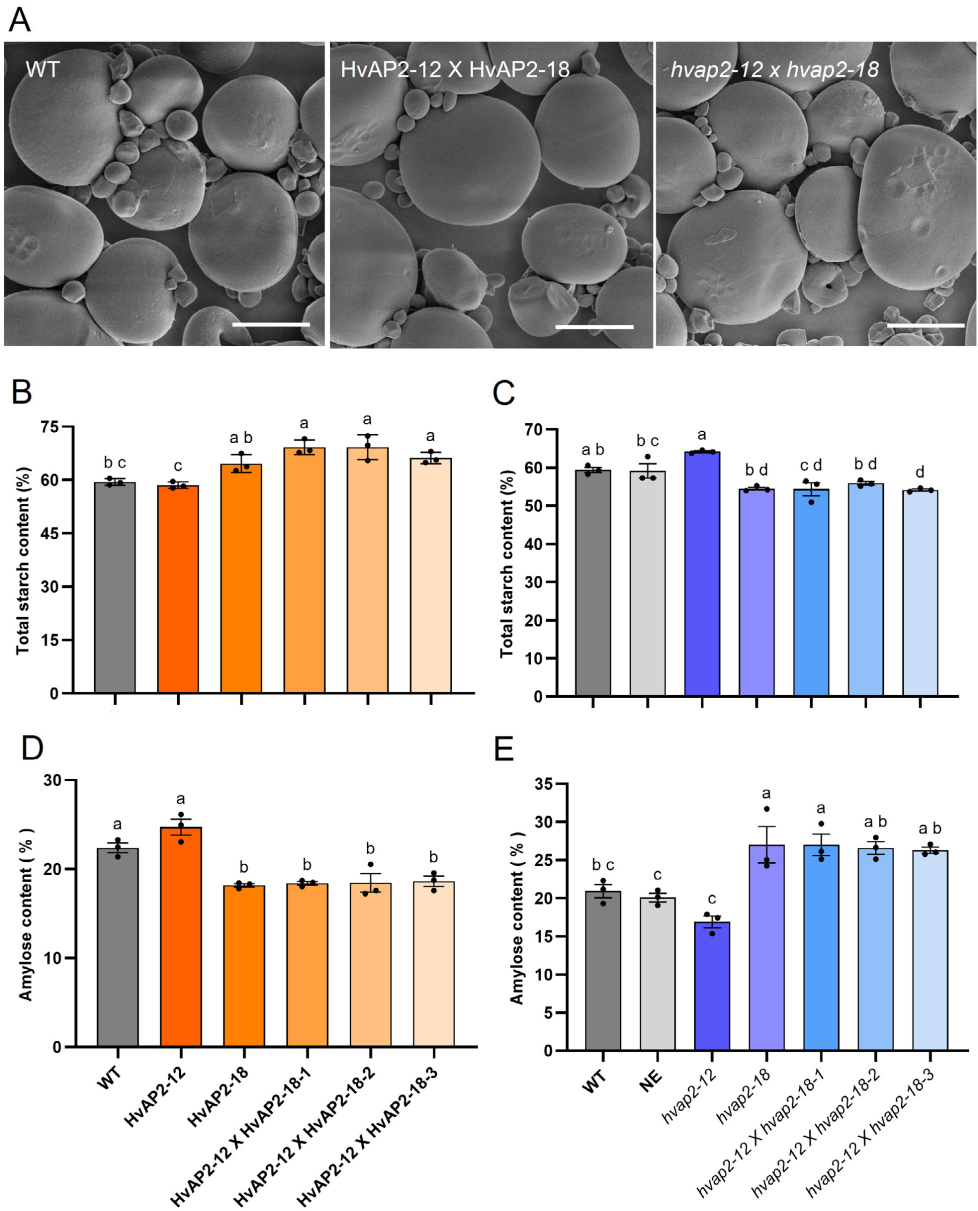
Double overexpression and mutant of *HvAP2‐12* and *HvAP2‐18* on starch content. (A) SEM of starch granules in the mature grain endosperm of *HvAP2‐12* and *HvAP2‐18* overexpression, mutant and WT lines. Scale bar: 10 μm. (B, C) Total starch content in HvAP2‐12, HvAP2‐18 and HvAP2‐12 × HvAP2‐18 double overexpression lines compared to WT (B); total starch content in *hvap2‐12*, *hvap2‐18* and *hvap2‐12 × hvap2‐18* double mutant lines compared to WT (C). (D, E) Amylose content in HvAP2‐12, HvAP2‐18 and HvAP2‐12 × HvAP2‐18 double overexpression lines compared to WT (D); amylose content in *hvap2‐12*, *hvap2‐18* and *hvap2‐12 × hvap2‐18* double mutant lines compared to WT. (E). Data are presented as mean ± SD from three biological replicates. Values with different letters indicate significant differences, as determined by one‐way ANOVA with Tukey's post hoc test (*p* < 0.05).

**FIGURE 9:**
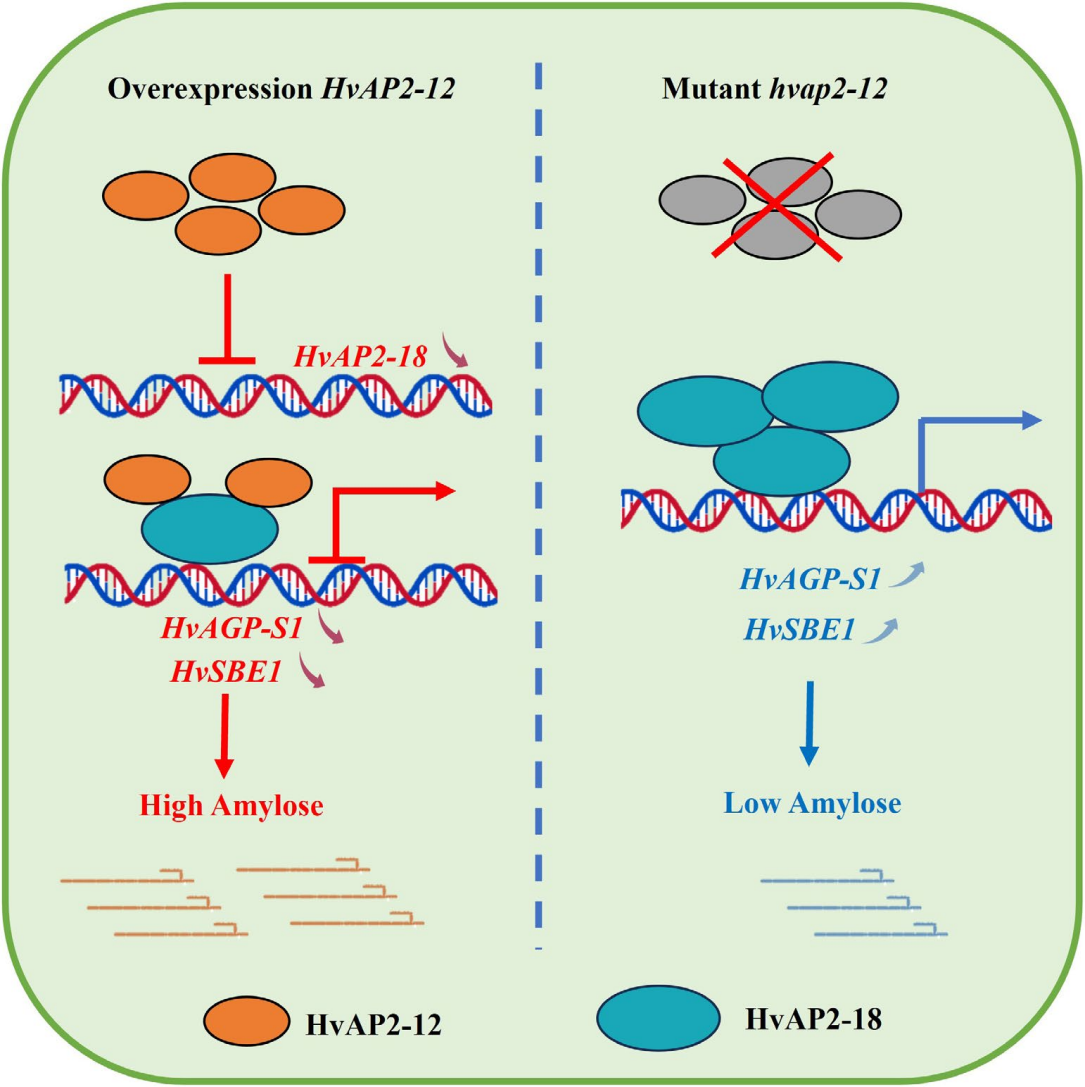
Model of HvAP2‐12 inhibiting HvAP2‐18‐mediated regulation of starch synthesis. Left: In *HvAP2‐12* overexpression lines, HvAP2‐12 binds to the HvAP2‐18 promoter to inhibit its expression; meanwhile, the heterodimer formed by HvAP2‐12 and HvAP2‐18 impairs the activation ability of starch synthase gene promoters *HvAGP‐S1* and *HvSBE1*, thus suppressing starch synthesis. Right: In *hvap2‐12* mutants, loss of HvAP2‐12‐mediated suppression releases *HvAP2‐18*'s repression on *HvAGP‐S1* and *HvSBE1* transcription, restoring starch synthesis.

SEM revealed no starch granule morphology differences among WT, double OE and double KO lines (Figure 8A), with consistent granule size distributions (Figure S26), suggesting these genes do not influence starch architecture. Starch profiling showed elevated total starch and reduced amylose in double OE lines (matching HvAP2‐18 OE trends; Figure 8B,C), while double mutants displayed decreased total starch and increased amylose (similar to *hvap2‐18* mutants; Figure 8D,E). These results strongly support *HvAP2‐12* acting upstream of *HvAP2‐18* in starch composition regulation (Figure 9).

## 3 Discussion

### 3.1 Regulatory Interaction Between *HvAP2‐12* and *HvAP2‐18* in Barley Starch Biosynthesis

Our study unveils a novel dual‐layer regulatory paradigm in barley starch biosynthesis, where *HvAP2‐12* indirectly governs starch composition through hierarchical repression of *HvAP2‐18*. Unlike direct transcriptional activators, *HvAP2‐12* employs a two‐tiered strategy: it transcriptionally suppresses *HvAP2‐18* expression via promoter binding, while concurrently inhibiting its DNA‐binding capacity through protein–protein interactions (Figure 9). This integrated mechanism combining transcriptional repression with non‐degradative post‐translational interference represents an unprecedented innovation within the AP2/ERF family, contrasting sharply with singular regulatory modes in other cereals. For instance, maize *ZmNAC128/130* relies on cooperative synergy for starch‐protein balance (Chen et al. 2023), whereas rice *OsNAC25* antagonises *OsNAC20/26* through unidirectional repression (Wang et al. 2024). Our work thus establishes the first AP2‐based cascade for metabolic fine‐tuning, highlighting species‐specific adaptation in barley.

The functional divergence between *HvAP2‐12* and *HvAP2‐18* resolves key phenotypic contradictions and underscores precise metabolic control. *HvAP2‐18* acts as the primary executor, directly activating starch synthesis genes (e.g., *HvAGP‐S1* and *HvSBE1*) to enhance amylopectin production and total starch content. In stark contrast, *HvAP2‐12* serves solely as a meta‐regulator, fine‐tuning amylose/amylopectin ratios without altering total starch levels or granule morphology, a specificity that is absent in maize or rice orthologues. This dichotomy explains the paradox where *HvAP2‐12* overexpression downregulates *HvAGP‐L/S1* but maintains starch homeostasis; compensatory pathways (e.g., alternative synthase activation) buffer flux disruptions, ensuring architectural integrity while permitting compositional adjustment (Kang et al. 2013). Such metabolic insulation, where *HvAP2‐12* modulates flux without structural perturbation, distinguishes our model from NAC‐dominated systems that often impact broader traits like grain size.

The *HvAP2‐12/18* axis offers transformative potential for barley quality engineering but reveals critical gaps for future exploration. By enabling stoichiometric control of starch composition, this mechanism allows targeted manipulation, including amylose enhancement for slow‐digesting functional foods and amylopectin boosting for industrial adhesives, while fully maintaining granule architecture integrity. However, unresolved questions persist: the stoichiometric dynamics of their protein interactions remain undefined, and compensatory pathways (e.g., sucrose transporter upregulation) that maintain starch homeostasis under *HvAP2‐12* suppression require mechanistic elucidation. Collectively, this dual‐layer transcriptional‐protein regulation by *HvAP2‐12* fine‐tunes starch synthesis through repression of *HvAP2‐18* activity, establishing a novel paradigm in cereal metabolic engineering.

### 3.2 *HvAP2‐12* and *HvAP2‐18* as Candidate Genes for Improving Barley Processing Quality

Starch constitutes 50%–70% of barley grain dry weight and is its most critical component. Starch content affects grain weight, which in turn influences yield, while starch composition and structure impact physicochemical properties, hydrolysis characteristics and ultimately determine barley's nutritional and processing quality (Hu et al. 2021; Lockyer and Nugent 2017). In this study, total starch content significantly increased in *HvAP2‐12* mutant lines, while no significant differences in 100‐grain weight were observed compared to WT. Conversely, *HvAP2‐18* overexpression lines exhibited significant increases in both total starch content and 100‐grain weight compared to WT. Interestingly, while total starch content in double‐gene overexpression lines exceeded WT levels, it remained comparable to *HvAP2‐18* overexpression lines. This finding highlights the dominant influence of HvAP2‐18 on starch accumulation, suggesting that *HvAP2‐12* primarily inhibits starch content through its interaction with *HvAP2‐18*.

The observed increase in total starch content, particularly driven by *HvAP2‐18*, holds direct implications for industrial applications. Elevated starch content could enhance ethanol yield in fermentation processes (Aydemir et al. 2014) and improve the energy density of barley‐based animal feeds. Furthermore, the altered amylose/amylopectin ratio in mutant lines suggests potential for tailoring starch functionality—higher amylose content is associated with slower digestibility and resistant starch formation, which could benefit diabetic food formulations, while amylopectin‐rich starches are preferred for adhesive production and brewing (Li et al. 2022). *HvAP2‐18*‐enhanced lines for bioethanol production where high starch yield is critical, and *HvAP2‐12/HvAP2‐18* co‐regulated lines with customised amylose content for functional foods or biodegradable plastics.

Future research must bridge mechanistic discovery with agricultural translation through integrated strategies: structural dissection of *HvAP2‐12/18* interaction interfaces will enable engineering of variants for precision metabolic control. Multi‐environment field trials across diverse agroclimatic zones must validate yield stability and starch functionality under abiotic stresses; ultimately deploying haplotype‐based genomic selection to accelerate development of elite cultivars with tailored starch architectures, thereby transforming regulatory insights into barley breeding pipelines.

## 4 Materials and Methods

### 4.1 Plant Materials

Barley cv. “Golden Promise” was used as the transgenic donor plant. Barley transformation was carried out as described by Harwood (2014); additional procedural details were adapted from a recent protocol (Jiang et al. 2023).

### 4.2 Subcellular Localisation

For wheat protoplasts, *HvAP2‐12* and *HvAP2‐18* coding regions were cloned into pJIT163‐GFP vectors and introduced into barley protoplasts via polyethylene glycol (PEG) mediation (Yoo et al. 2007).

### 4.3 Yeast One/Two‐Hybrid

The Yeast Hybrid assay was conducted following the protocol of the Matchmaker Yeast Hybrid Library Screening System (Clontech, USA).

### 4.4 RT‐qPCR

qRT‐PCR analysis was conducted on the CFX 96 Real‐Time System (Bio‐Rad) using ChamQ Universal SYBR qPCR Master Mix (Vazyme Biotech, Q121‐02/03).

### 4.5 Dual‐Luciferase Transcriptional Activity Assay

The pGreenII 62‐SK and pGreenII 0800‐LUC vectors were used for dual‐luciferase reporter assays. Firefly and Renilla luciferase activities were quantified using the Dual‐Luciferase Reporter Assay Kit (Vazyme Biotech) and a GloMax 96‐microwell plate luminescence detector (Promega).

### 4.6 Electrophoretic Mobility Shift Assay

Electrophoretic mobility shift assay (EMSA) was performed using the LightShift Chemiluminescent EMSA Kit (Pierce, USA).

### 4.7 Transcriptome Deep Sequencing (RNA‐Seq)

Sequencing libraries were prepared using the NEBNext Ultra RNA Library Prep Kit for Illumina (NEB, Ipswich, MA, USA) following the manufacturer's recommendations. Clustering of the index‐coded samples was performed using the cBot Cluster Generation System with the TruSeq PE Cluster Kit v4‐cBot‐HS (Illumina).

### 4.8 Phenotypic Identification

Phenotypic identification includes: grain phenotype observation, determination of starch, scanning electron microscopy, starch XRD diffraction analysis and analysis of starch chain length distribution, detailed in Supporting Information.

### 4.9 Statistical Analysis

All experiments were conducted with at least three biological replicates. Data were presented as means ± standard deviation (SD). Differences between HvAP2‐12, HvAP2‐18 and wild‐type lines were analysed using Student's *t*‐test in SPSS v.20 (SPSS Inc., Chicago, USA), with significance set at *p* < 0.05.

Due to space limitations, the specific content of the materials and methods is detailed in Supporting Information.

The primers used in the experiments are all listed in Table S1.

## Author Contributions

**Jinjin Ding:** validation, formal analysis, data curation, investigation, writing – original draft. **Yulong Li:** formal analysis, methodology, resources and visualisation. **Jing Liu:** formal analysis, methodology, resources and visualisation. **Na Lin:** formal analysis, methodology, resources, visualization. **David Seung:** formal analysis, methodology, resources and visualisation. **Qiang Xu:** formal analysis, methodology, resources and visualisation. **Yazhou Zhang:** methodology, software and writing – review and editing. **Huaping Tang:** methodology, software and writing – review and editing. **Pengfei Qi:** methodology, software and writing – review and editing. **Mei Deng:** methodology, visualisation and writing – review and editing. **Jian Ma:** methodology, visualisation and writing – review and editing. **Guoyue Chen:** methodology, visualisation and writing – review and editing. **Jirui Wang:** methodology, visualisation and writing – review and editing. **Yuming Wei:** writing – review and editing. **Qiantao Jiang:** conceptualisation, supervision, funding acquisition, project administration and writing – review and editing.

## Conflicts of Interest

The authors declare no conflicts of interest.

## Supporting information


**Table S1:** pbi70338‐sup‐0001‐TableS1.xlsx.

**Figure S1:** Heatmap analysis of the transcriptome data of the barley AP2 subfamily.
**Figure S2:** Structural prediction of the HvAP2‐12 protein.
**Figure S3:** Subcellular localisation of HvAP2‐12 in *Nicotiana benthamiana* leaves.
**Figure S4:** Gene editing sites for *HvAP2‐12* knockout mutants.
**Figure S5:** *HvAP2‐12* and *HvAP2‐18* promoter activity verified by GUS staining. Positive control: 1302‐GFP, CK‐: Plasmid free only buffer injection into tobacco.
**Figure S6:** *HvAP2‐12* expression in *HvAP2‐12* overexpression lines.
**Figure S7:** Analysis of grain morphology in *HvAP2‐12* overexpression and mutant lines. A–D. Morphological observations of plants and grains from *HvAP2‐12* overexpression, mutant and WT lines. E‐H. Average hundred‐grain weight (E), bulk density (F), grain length (G) and width (H) of overexpression, mutant and WT lines. Bulk density represents grain weight per litre of container volume, measured under standardised filling conditions. Data are presented as mean ± SD from three biological replicates. Values with different letters indicate significant differences, as determined by one‐way ANOVA with Tukey's post hoc test (*p* < 0.05).
**Figure S8:** Starch crystallinity quantification in *HvAP2‐12* transgenic barley. Data are presented as mean ± SD from three biological replicates. Values with different letters indicate significant differences, as determined by one‐way ANOVA with Tukey's post hoc test (*p* < 0.05).
**Figure S9:** Transcriptome analysis of HvAP2‐12. (A) *HvAP2‐12* overexpression, mutation and wild‐type lines samples were distributed, with each type being three independent lines. (B) volcano plot of differentially expressed genes.
**Figure S10:** Interaction between HvAP2‐12 and starch synthase gene DLR was verified.
**Figure S11:** Heatmap showing the expression levels of transcription factors that regulate grain starch have been reported in *HvAP2‐12* overexpression, knockout and WT lines.
**Figure S12:** Relative expression levels of *HvAP2‐18*, *MADS7*, *MADS14*, *MADS29*, *MADS56* and *RISBZ2* in *HvAP2‐12* overexpression and mutant lines compared to WT, estimated by RT‐qPCR and normalised to barley *β‐actin* and *HvGAPDH*. Data are presented as mean ± SD from three biological replicates. Values with different letters indicate significant differences, as determined by one‐way ANOVA with Tukey's post hoc test (*p* < 0.05).
**Figure S13:** Dual‐luciferase transcriptional activity assays to assess the effect of *HvAP2‐12* on *MADS7*, *MADS14*, *MADS29*, *MADS56* and *RISBZ2* expression. Values with different letters indicate significant differences, as determined by one‐way ANOVA with Tukey's post hoc test (*p* < 0.05).
**Figure S14:** Subcellular localisation of HvAP2‐18 in *Nicotiana benthamiana* leaves.
**Figure S15:** *HvAP2‐18* expression in *HvAP2‐18* overexpression lines.
**Figure S16:** Gene editing sites for *HvAP2‐18* knockout mutants.
**Figure S17:** Morphology of *HvAP2‐18* overexpression, mutant lines and WT plants.
**Figure S18:** Comparison of A‐ and B‐type starch granule content between *HvAP2‐18* overexpression and mutant lines and WT. (A) Granule size distributions were determined using a Coulter counter, with data expressed as relative % volume (of total starch) versus granule diameter. (B) B‐type granule volume (% of total starch) was extracted from the relative volume versus diameter plots by fitting a bimodal mixed normal distribution. (C) A‐type granule volume.
**Figure S19:** Analysis of the physicochemical properties of starch in *HvAP2‐18* overexpression and mutant lines compared to WT. (A) X‐ray diffraction (XRD) analysis of starch from *HvAP2‐18*, HvAP2‐18‐8 and hvap2‐18‐63 lines were selected for the determination. (B) Distribution of amylopectin chain length in *HvAP2‐18*, the selected lines are the same as in A.
**Figure S20:** Transcriptome and expression analysis of *HvAP2‐18*. (A, B) Venn diagram of *HvAP2‐18* differential genes. Set up three comparison groups: WT versus HvAP2‐18, WT versus *hvap2‐18*, HvAP2‐18 versus *hvap2‐18*. (B) *HvAP2‐18* transcriptome data differential gene scatter plot. The HvAP2‐18 versus *hvap2‐18* up‐regulates and then downregulates genes. (C, D) GO enrichment and KEGG pathway analysis for *HvAP2‐18* overexpression and mutant lines compared to WT.
**Figure S21:** Relative expression levels of starch and sugar genes in *HvAP2‐18* overexpression and mutants lines compared to WT, estimated by RT‐qPCR and normalised to barley *β‐actin* and *HvGAPDH*.
**Figure S22:** *HvAP2‐18 binds to the HvAGP‐S1 promoter‐1* and *HvSBE1 promoter‐3*. (A) Schematic representation of the dual‐luciferase reporter (DLR) assay constructs, showing the *HvAGP‐S* and *HvSBE1* promoter segments and HvAP2‐18 binding vectors. (B, C) DLR assays confirming *HvAP2‐18* binding to promoter fragments of the starch synthase genes *HvAGP‐S* and *HvSBE1*.
**Figure S23:** Prediction of the 1500 bp upstream promoter element of *HvAGP‐S1* and *HvSBE1*.
**Figure S24:** Relative expression levels of *HvAP2‐12* and *HvAP2‐18* in *HvAP2‐12* overexpression *HvAP2‐18* overexpression and double overexpression compared to WT, estimated by RT‐qPCR and normalised to barley *β‐actin* and *HvGAPDH*. Red represents the expression level of *HvAP2‐12*, and blue represents the expression level of *HvAP2‐18*. Data are presented as mean ± SD from three biological replicates. Values with different letters indicate significant differences, as determined by one‐way ANOVA with Tukey's post hoc test (*p* < 0.05).
**Figure S25:** Analysis of grain morphology in double overexpression and mutant lines of *HvAP2‐12* and *HvAP2‐18*.
**Figure S26:** Analysis of starch granule content in double overexpression and mutant lines of *HvAP2‐12* and *HvAP2‐18*.

**Data S1:** pbi70338‐sup‐0003‐Supinfo.docx.

## Data Availability

The data that support the findings of this study are available on request from the corresponding author. The data are not publicly available due to privacy or ethical restrictions.
