# Supplementary material for: Dual‐Layer Transcriptional‐Protein Regulation by HvAP2‐12 Represses HvAP2‐18 Activity to Fine‐Tune Barley Starch Synthesis
Source: Plant Biotechnol J. 2025 Aug 23;23(12):5694–709. doi: 10.1111/pbi.70338 (PMC12665055; doi:10.1111/pbi.70338)
Supplement: Supplementary file 2 — Figure S1: Heatmap analysis of the transcriptome data of the barley AP2 subfamily. Figure S2: Structural prediction of the HvAP2‐12 protein. Figure S3: Subcellular localisation of HvAP2‐12 in Nicotiana benthamiana leaves. Figure S4: Gene editing sites for HvAP2‐12 knockout mutants. Figure S5: HvAP2‐12 and HvAP2‐18 promoter activity verified by GUS staining. Positive control: 1302‐GFP, CK‐: Plasmid free only buffer injection into tobacco. Figure S6: HvAP2‐12 expression in HvAP2‐12 overexpression lines. Figure S7: Analysis of grain morphology in HvAP2‐12 overexpression and mutant lines. A–D. Morphological observations of plants and grains from HvAP2‐12 overexpression, mutant and WT lines. E‐H. Average hundred‐grain weight (E), bulk density (F), grain length (G) and width (H) of overexpression, mutant and WT lines. Bulk density represents grain weight per litre of container volume, measured under standardised filling conditions. Data are presented as mean ± SD from three biological replicates. Values with different letters indicate significant differences, as determined by one‐way ANOVA with Tukey's post hoc test (p < 0.05). Figure S8: Starch crystallinity quantification in HvAP2‐12 transgenic barley. Data are presented as mean ± SD from three biological replicates. Values with different letters indicate significant differences, as determined by one‐way ANOVA with Tukey's post hoc test (p < 0.05). Figure S9: Transcriptome analysis of HvAP2‐12. (A) HvAP2‐12 overexpression, mutation and wild‐type lines samples were distributed, with each type being three independent lines. (B) volcano plot of differentially expressed genes. Figure S10: Interaction between HvAP2‐12 and starch synthase gene DLR was verified. Figure S11: Heatmap showing the expression levels of transcription factors that regulate grain starch have been reported in HvAP2‐12 overexpression, knockout and WT lines. Figure S12: Relative expression levels of HvAP2‐18, MADS7, MADS14, MADS29, MADS56 and RISBZ2 in H [file PBI-23-5694-s003.docx]

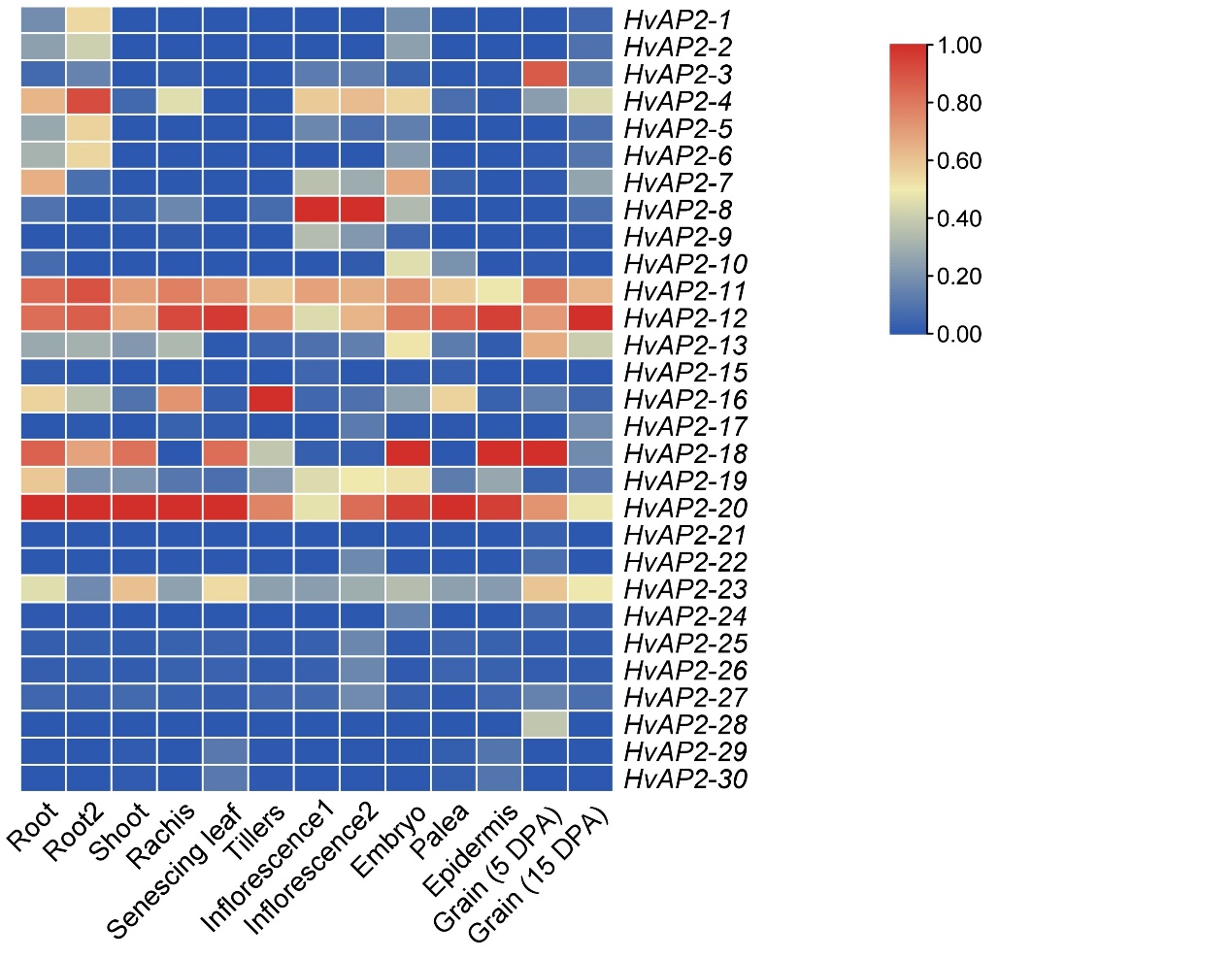


Figure S1. Heatmap analysis of the transcriptome data of the barley AP2 subfamily.


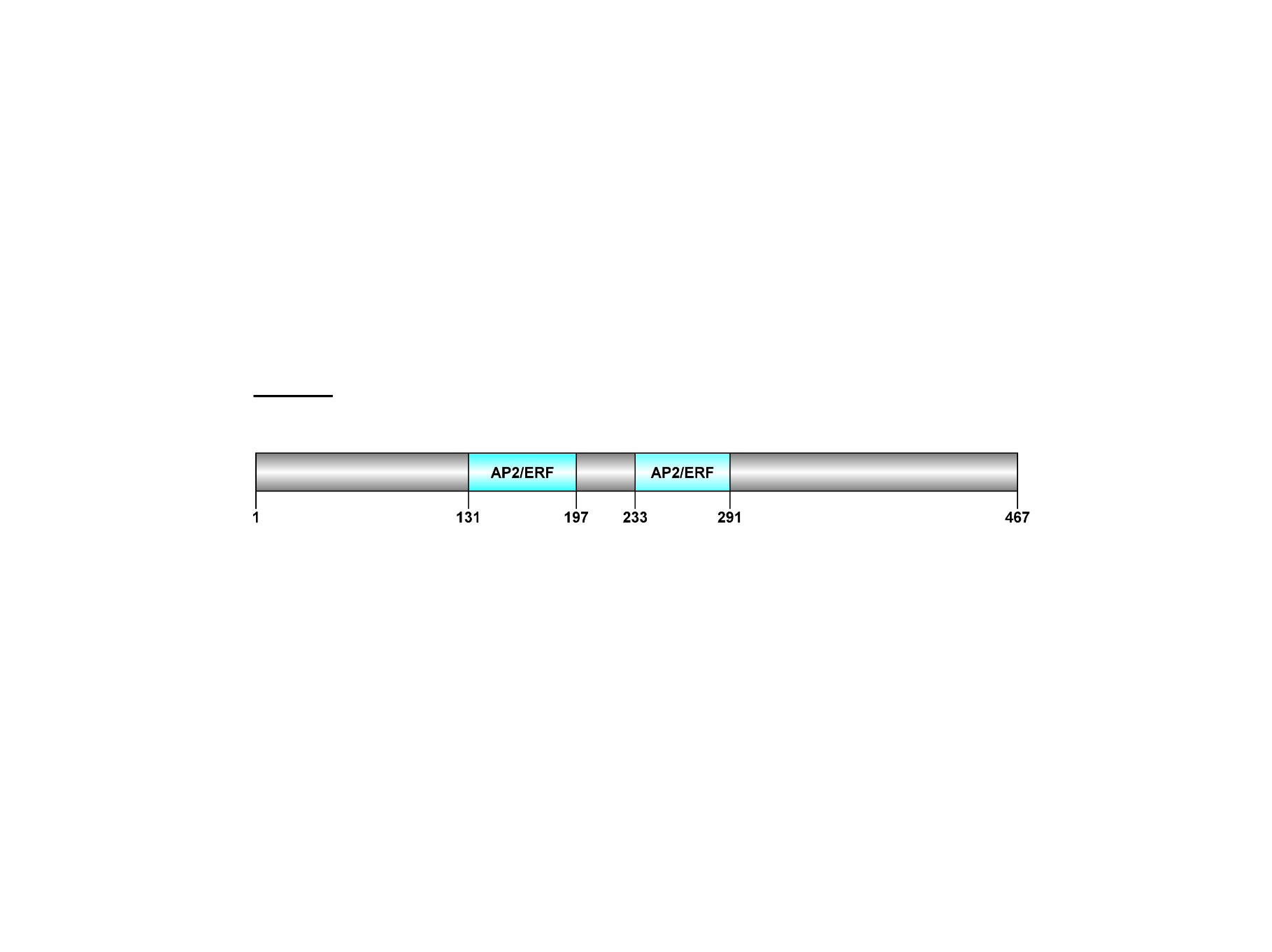


Figure S2. Structural prediction of the HvAP2-12 protein.


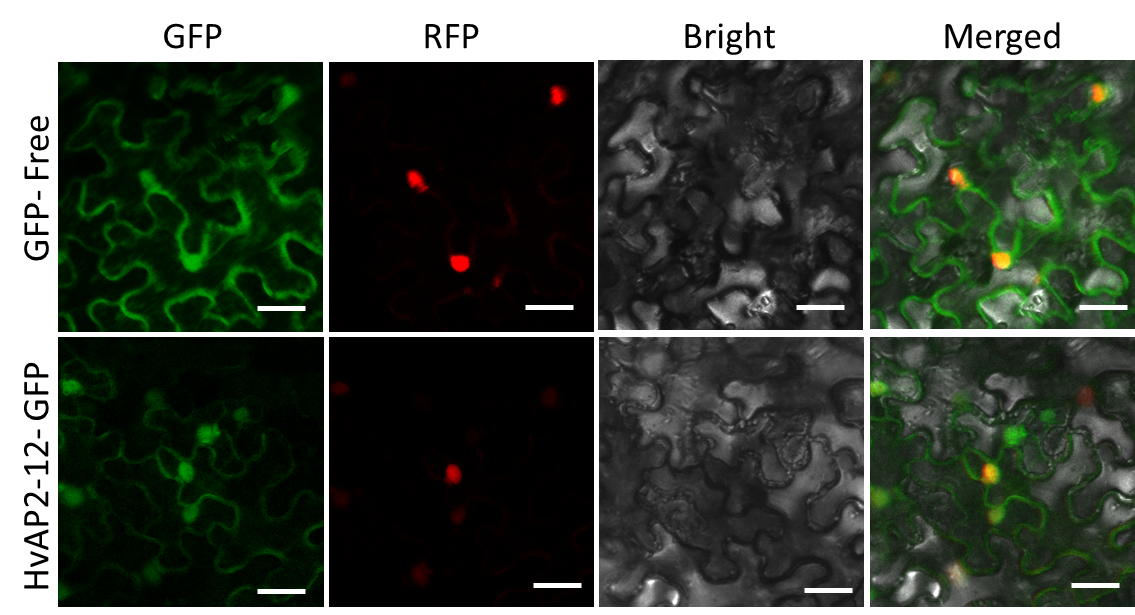
Figure S3. Subcellular localization of HvAP2-12 in *Nicotiana benthamiana* leaves.


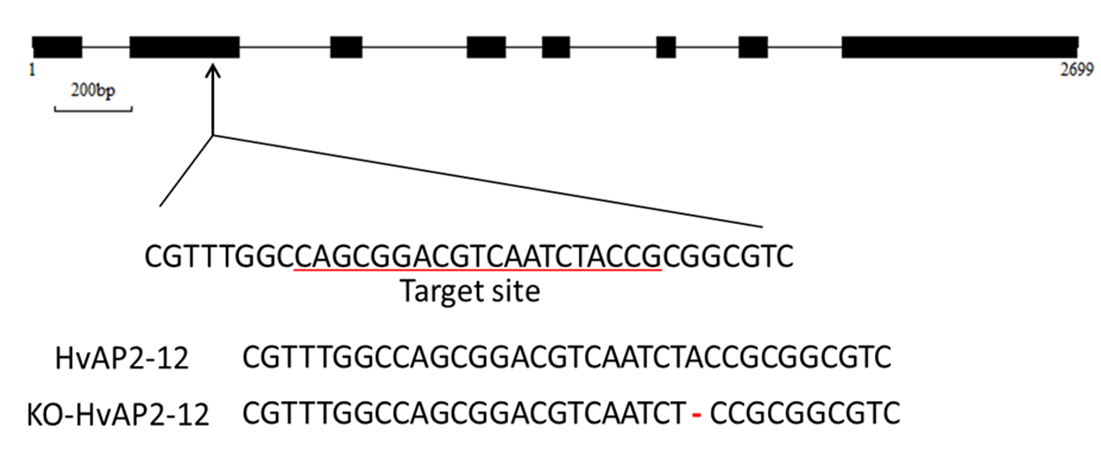


Figure S4. Gene editing sites for *HvAP2-12* knockout mutants.


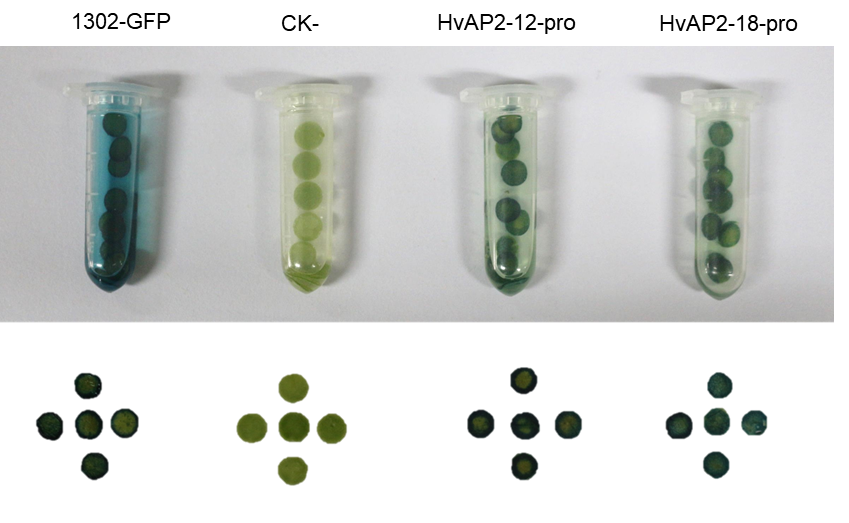


Figure S5. *HvAP2-12* and *HvAP2-18* promoter activity verified by GUS staining. Positive control: 1302-GFP, CK-: Plasmid free only buffer injection into tobacco.

Figure S6 *HvAP2-12* expression in *HvAP2-12* overexpression lines.


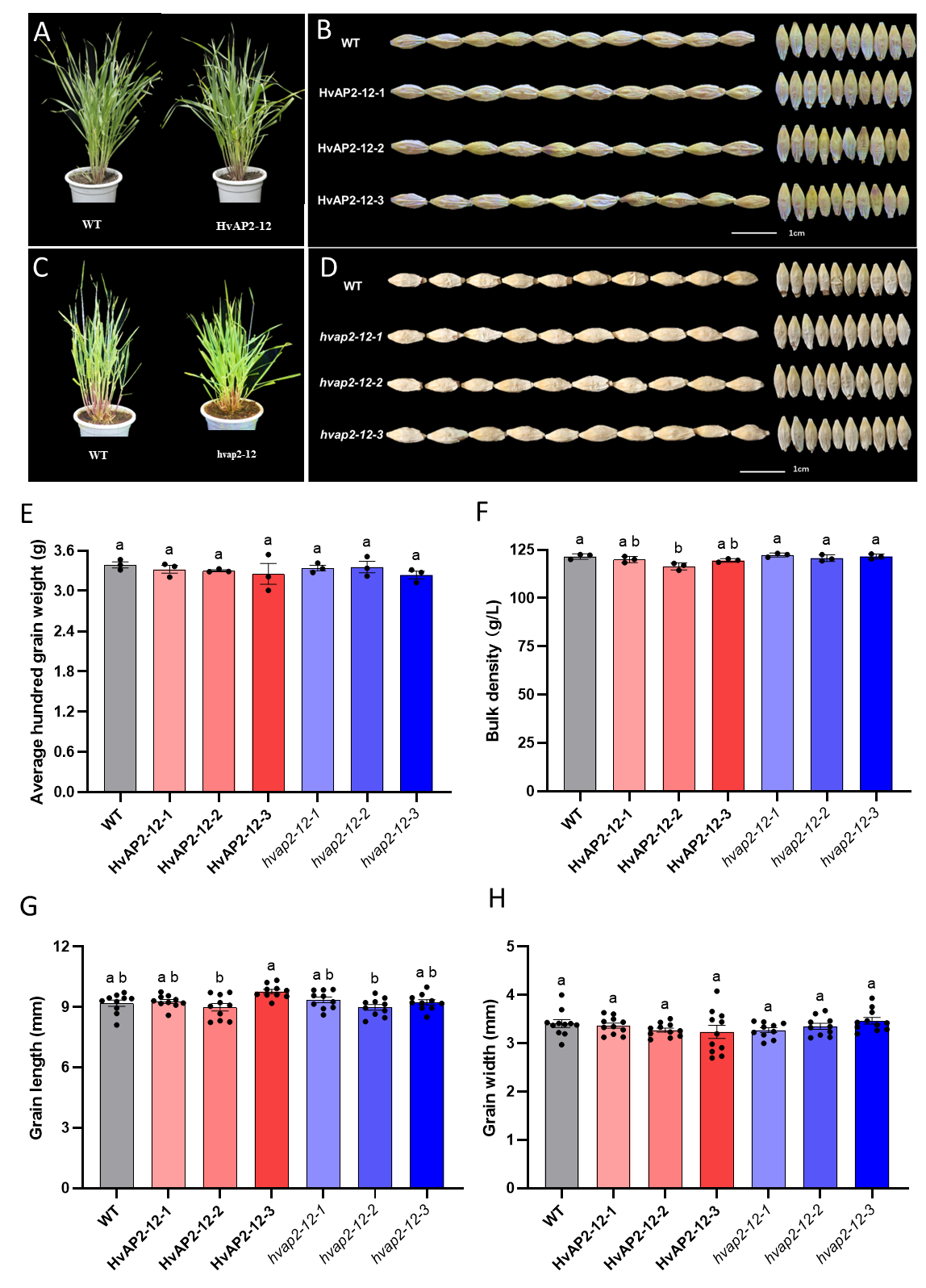


Figure S7. **Analysis of grain morphology in *HvAP2-12* overexpression and mutant lines.** A-D. Morphological observations of plants and grains from *HvAP2-12* overexpression, mutant, and WT lines. E-H. Average hundred-grain weight (E), bulk density (F), grain length (G), and width (H) of overexpression, mutant, and WT lines. Bulk density represents grain weight per liter of container volume, measured under standardized filling conditions. Data are presented as mean ± SD from three biological replicates. Values with different letters indicate significant differences, as determined by one-way ANOVA with Tukey’s post hoc test (*p* < 0.05).

**Figure S8 Starch crystallinity quantification in *HvAP2-12* transgenic barley.** Data are presented as mean ± SD from three biological replicates. Values with different letters indicate significant differences, as determined by one-way ANOVA with Tukey’s post hoc test (*p* < 0.05).


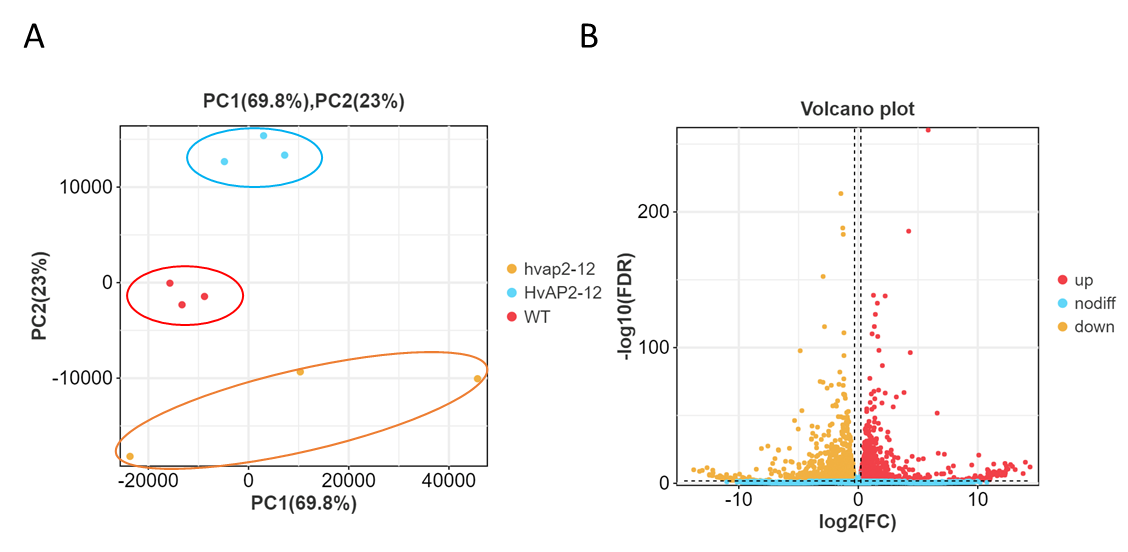


Figure S9. Transcriptome analysis of HvAP2-12. A. *HvAP2-12* overexpression, mutation, and wild-type lines samples were distributed, with each type being three independent lines. B. volcano plot of differentially expressed genes.

Figure S10. Interaction between HvAP2-12 and starch synthase gene DLR was verified.


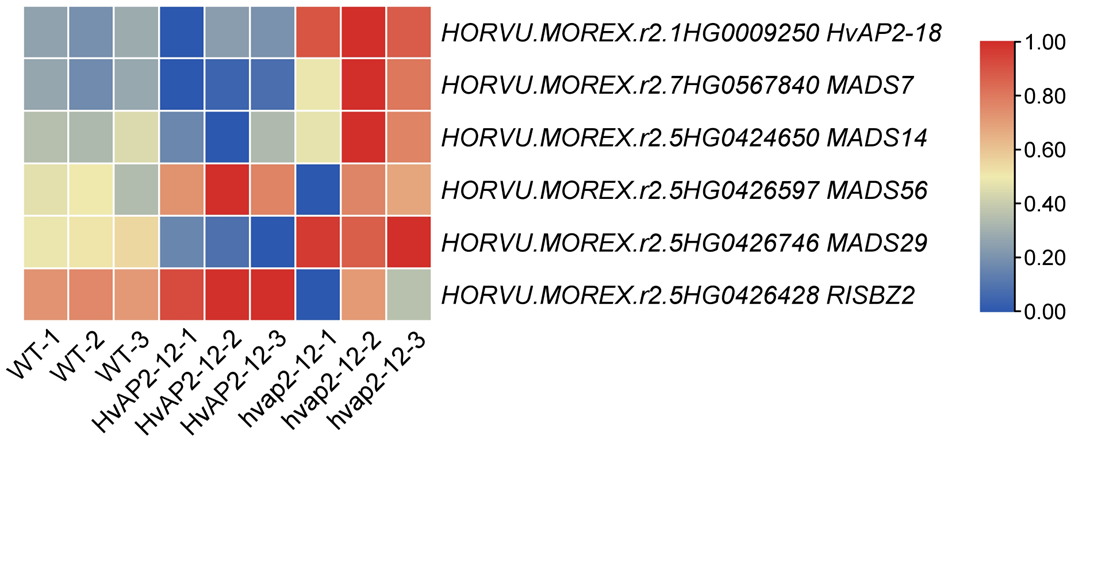


Figure S11. Heatmap showing the expression levels of transcription factors that regulate grain starch have been reported in *HvAP2-12* overexpression, knockout, and WT lines.

Figure S12. Relative expression levels of *HvAP2-18*, *MADS7*, *MADS14*, *MADS29*, *MADS56*, and *RISBZ2* in *HvAP2-12* overexpression and mutant lines compared to WT, estimated by RT-qPCR and normalized to barley *β-actin* and *HvGAPDH.* Data are presented as mean ± SD from three biological replicates. Values with different letters indicate significant differences, as determined by one-way ANOVA with Tukey’s post hoc test (*p* < 0.05).

Figure S13. Dual luciferase transcriptional activity assays to assess the effect of *HvAP2-12* on *MADS7*, *MADS14*, *MADS29*, *MADS56*, and *RISBZ2* expression. Values with different letters indicate significant differences, as determined by one-way ANOVA with Tukey’s post hoc test (*p* < 0.05).


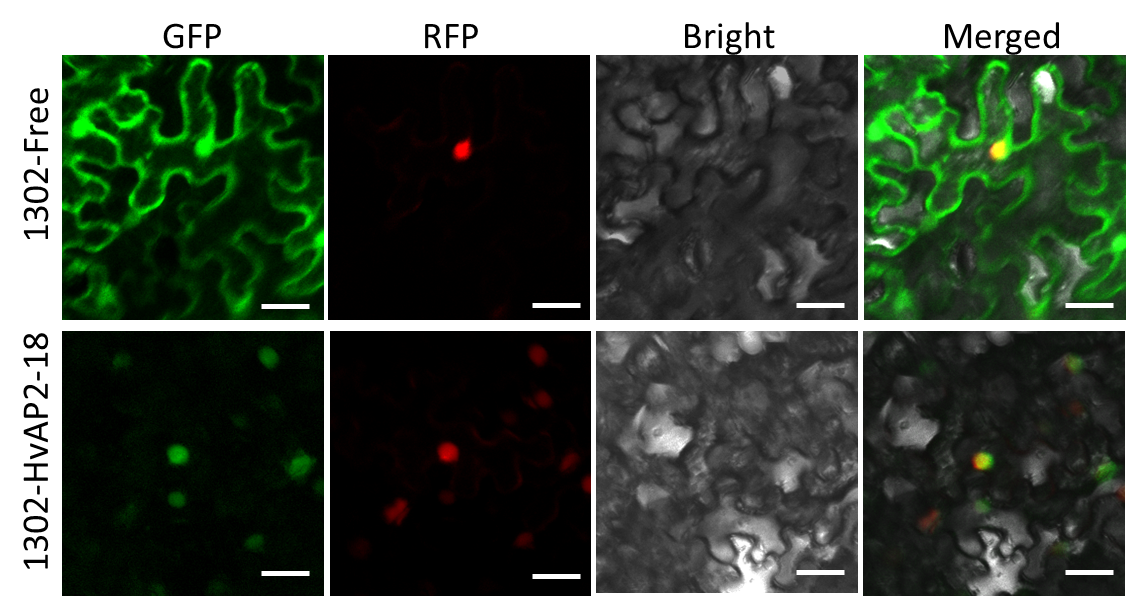


Figure S14. Subcellular localization of HvAP2-18 in *Nicotiana benthamiana* leaves.

Figure S15. *HvAP2-18* expression in *HvAP2-18* overexpression lines.


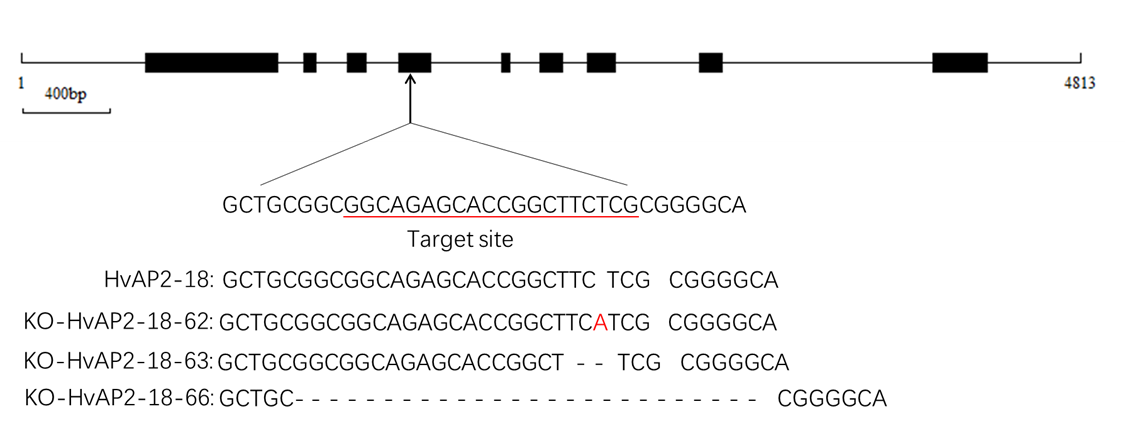


Figure S16. Gene editing sites for *HvAP2-18* knockout mutants.


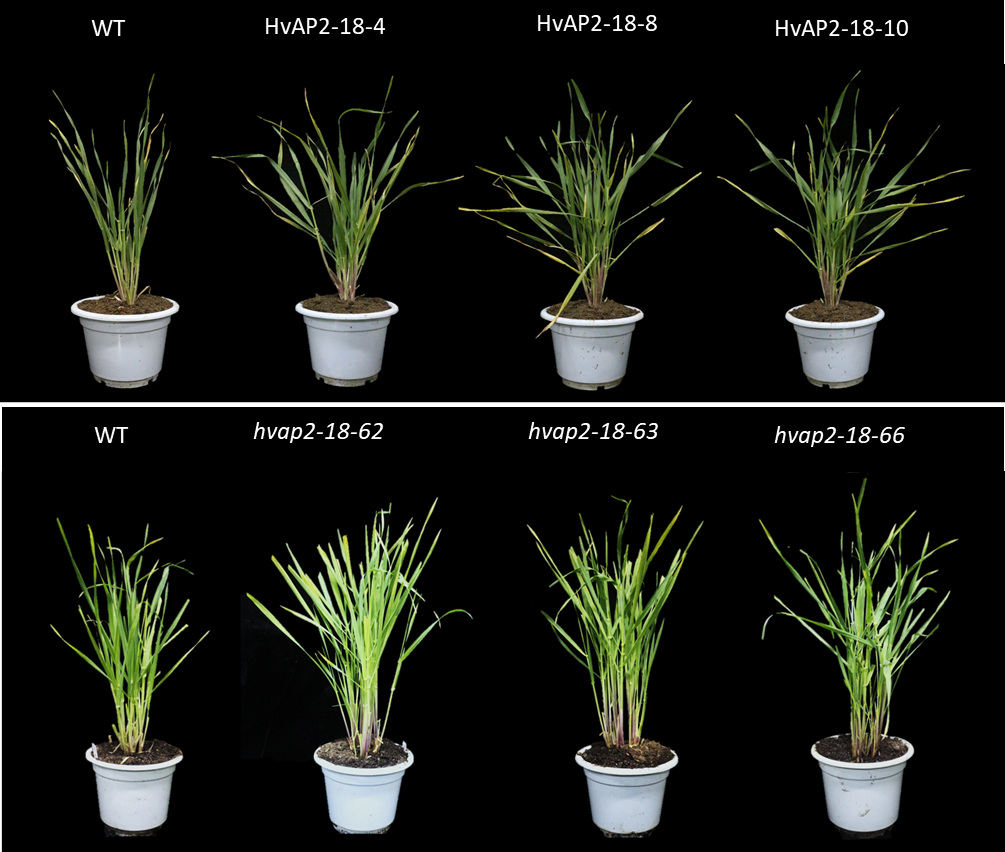


Figure S17. Morphology of *HvAP2-18* overexpression, mutant lines and WT plants.


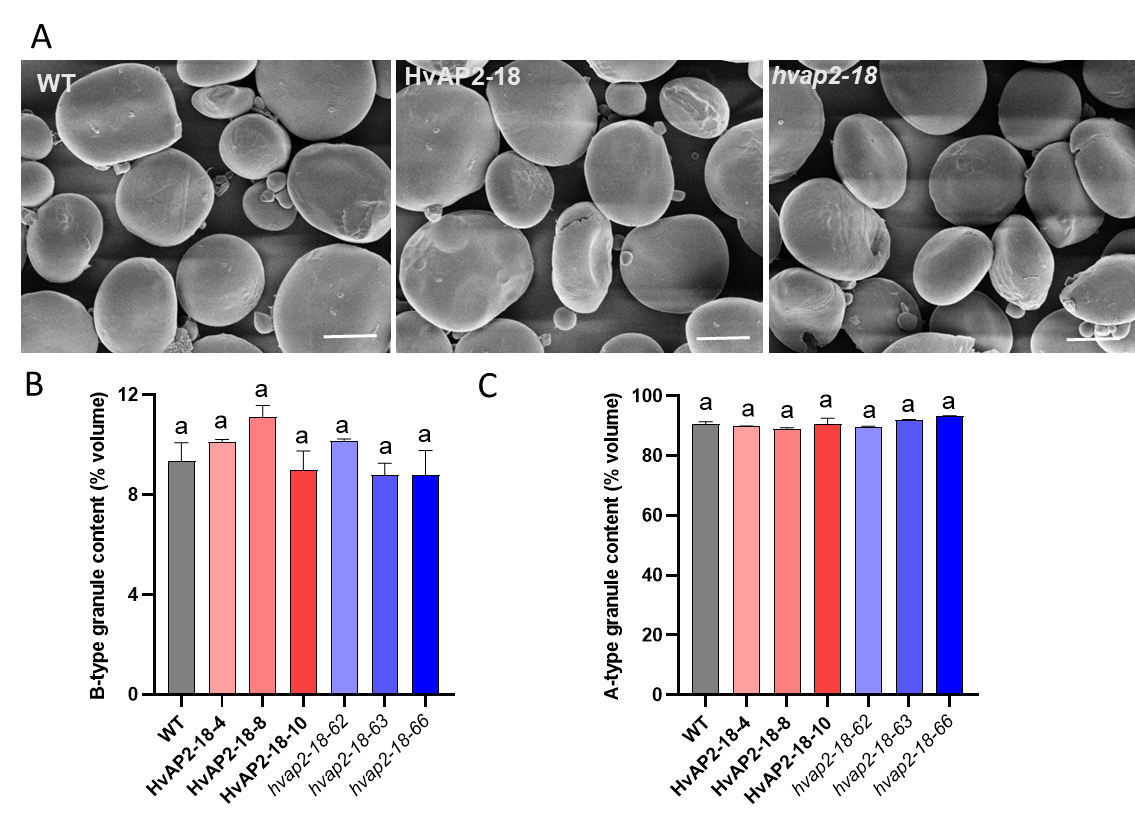


Figure S18. Comparison of A- and B-type starch granule content between *HvAP2-18* overexpression and mutant lines and WT. A. Granule size distributions were determined using a Coulter counter, with data expressed as relative % volume (of total starch) vs. granule diameter. B. B-type granule volume (% of total starch) was extracted from the relative volume vs. diameter plots by fitting a bimodal mixed normal distribution. C. A-type granule volume.


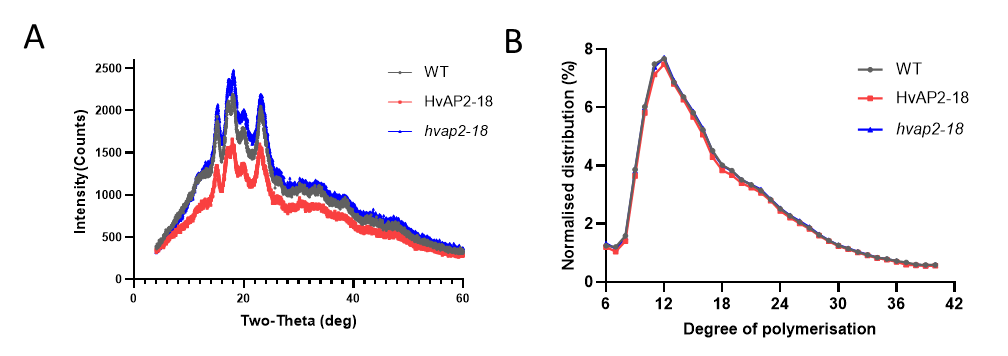


Figure S19. Analysis of the physicochemical properties of starch in *HvAP2-18* overexpression and mutant lines compared to WT. A. X-ray diffraction (XRD) analysis of starch from *HvAP2-18*, HvAP2-18-8 and hvap2-18-63 lines were selected for the determination. B. Distribution of amylopectin chain length in *HvAP2-18*, the selected lines are the same as in A).


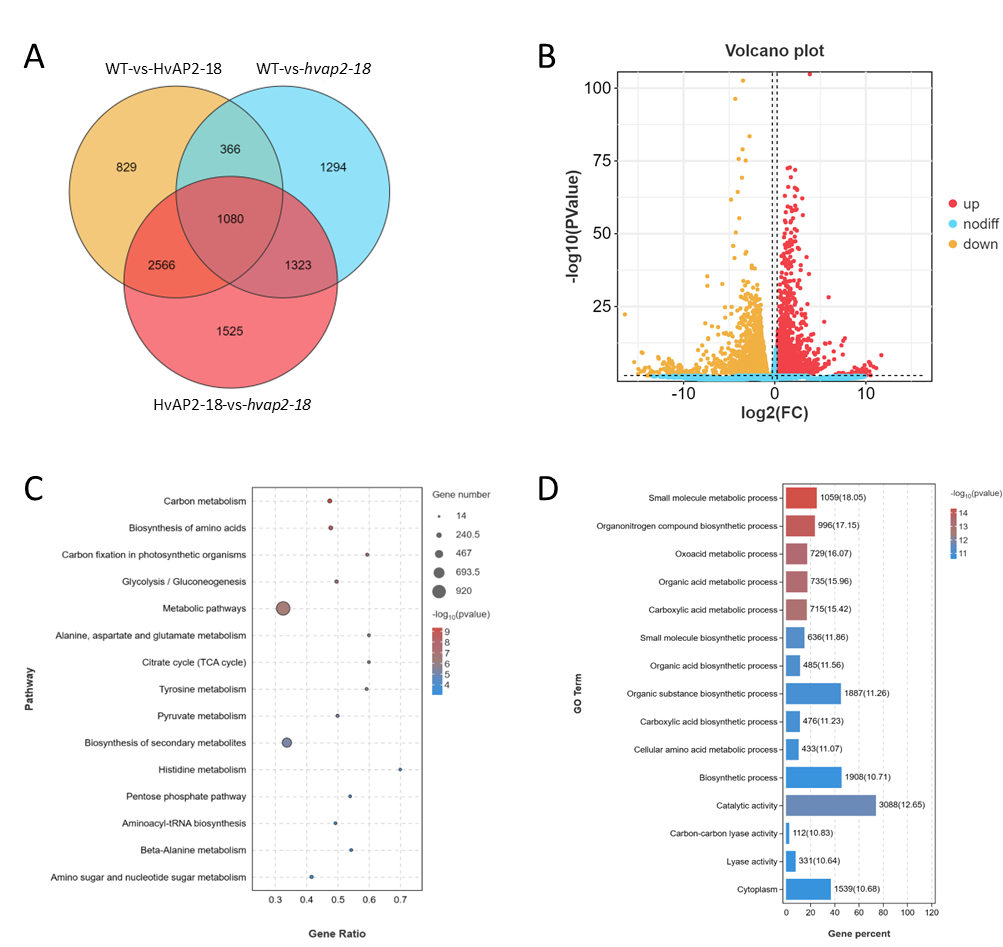


Figure S20. Transcriptome and expression analysis of *HvAP2-18*. A-B: Venn diagram of *HvAP2-18* differential genes. Set up three comparison groups: WT vs. HvAP2-18, WT vs. *hvap2-18*, HvAP2-18 vs. *hvap2-18*. B. *HvAP2-18* transcriptome data differential gene scatter plot. The HvAP2-18 vs. *hvap2-18* up-regulates and then down-regulates genes. C-D: GO enrichment and KEGG pathway analysis for *HvAP2-18* overexpression and mutant lines compared to WT.


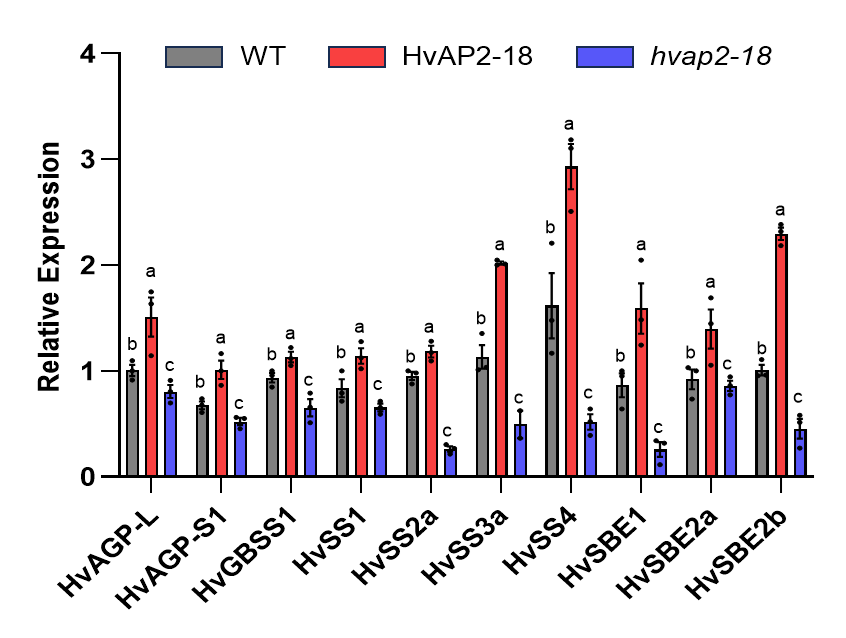


Figure S21. Relative expression levels of starch and sugar genes in *HvAP2-18* overexpression and mutants lines compared to WT, estimated by RT-qPCR and normalized to barley *β-actin* and *HvGAPDH.*


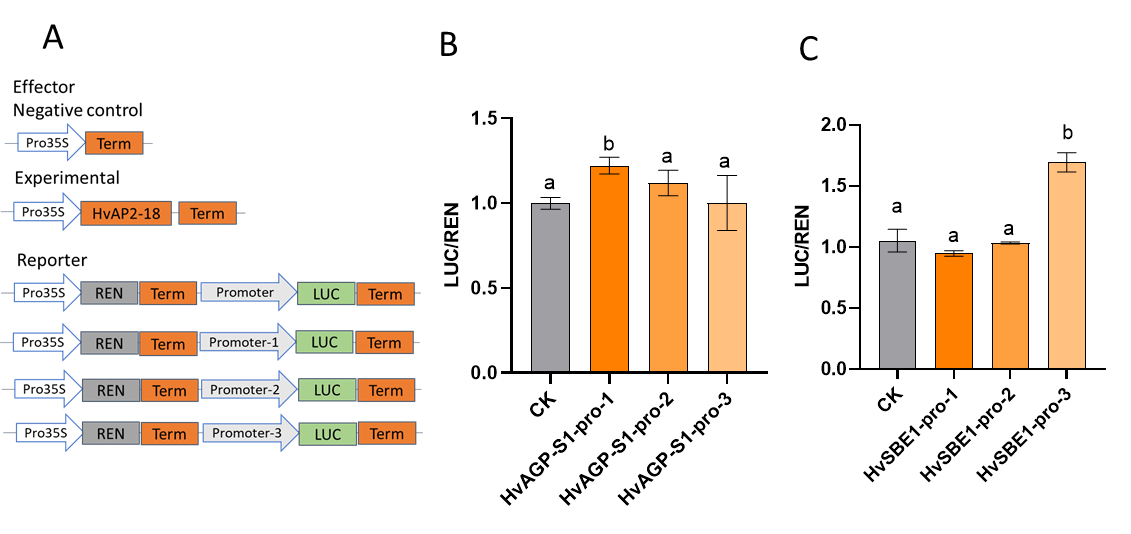


Figure S22. *HvAP2-18 binds to the HvAGP-S1 promoter-1* and *HvSBE1 promoter-3.* A. Schematic representation of the dual-luciferase reporter (DLR) assay constructs, showing the *HvAGP-S* and *HvSBE1* promoter segments and HvAP2-18 binding vectors. B-C. DLR assays confirming *HvAP2-18* binding to promoter fragments of the starch synthase genes *HvAGP-S* and *HvSBE1*.


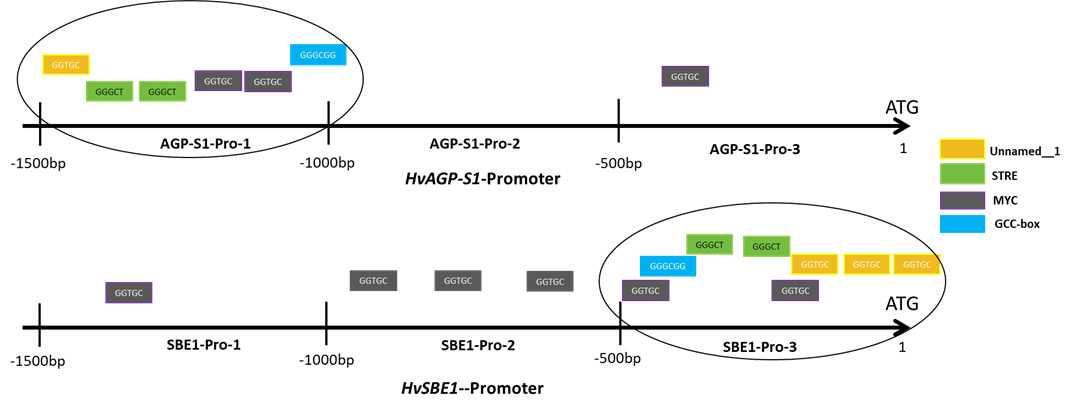


Figure S23. Prediction of the 1500 bp upstream promoter element of *HvAGP-S1* and *HvSBE1*.

Figure S24. Relative expression levels of *HvAP2-12* and *HvAP2-18* in *HvAP2-12* overexpression *HvAP2-18* overexpression and double overexpression compared to WT, estimated by RT-qPCR and normalized to barley *β-actin* and *HvGAPDH.* Red represents the expression level of *HvAP2-12*, and blue represents the expression level of *HvAP2-18*. Data are presented as mean ± SD from three biological replicates. Values with different letters indicate significant differences, as determined by one-way ANOVA with Tukey’s post hoc test (*p* < 0.05).


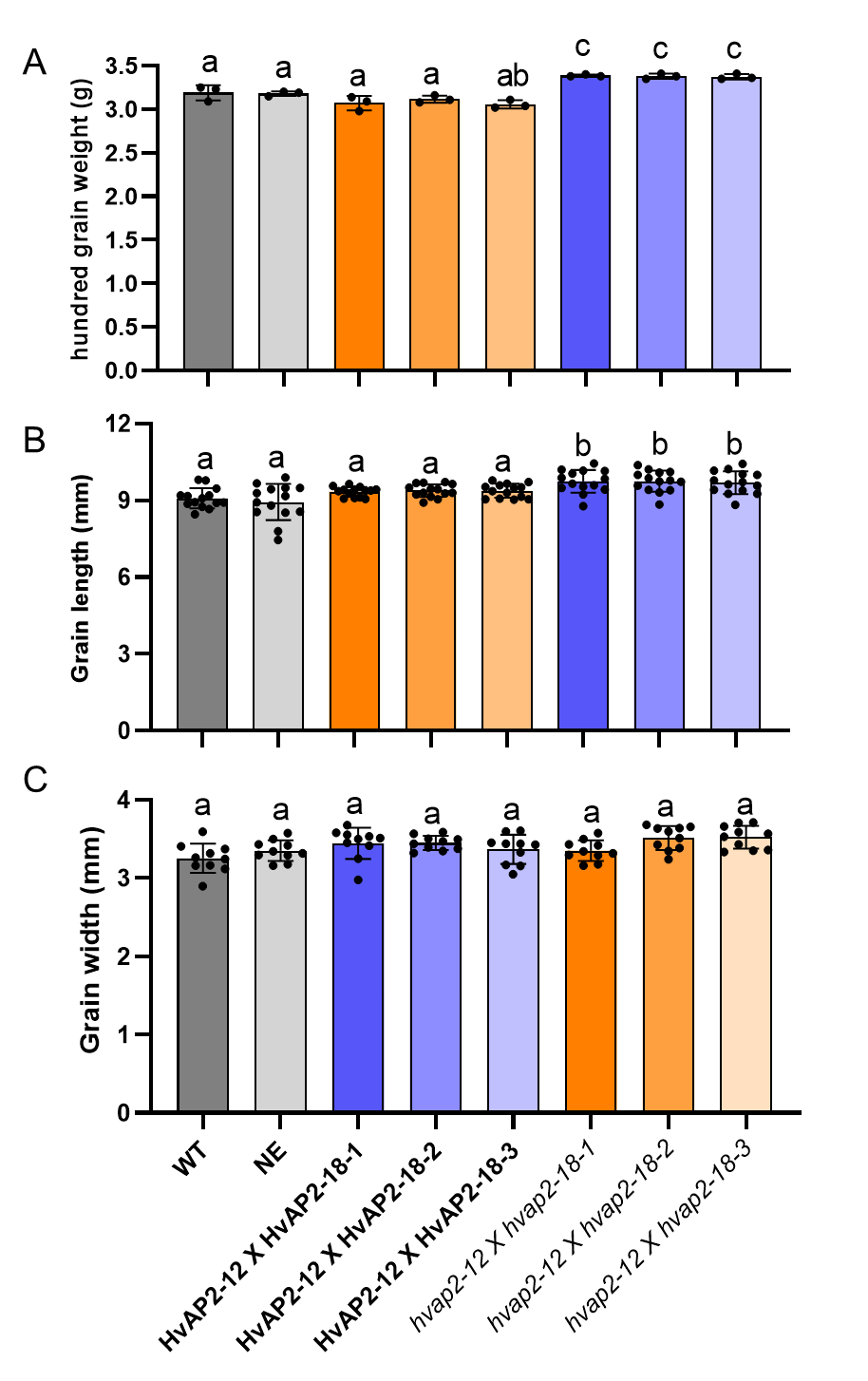


Figure S25. Analysis of grain morphology in double overexpression and mutant lines of *HvAP2-12* and *HvAP2-18*.


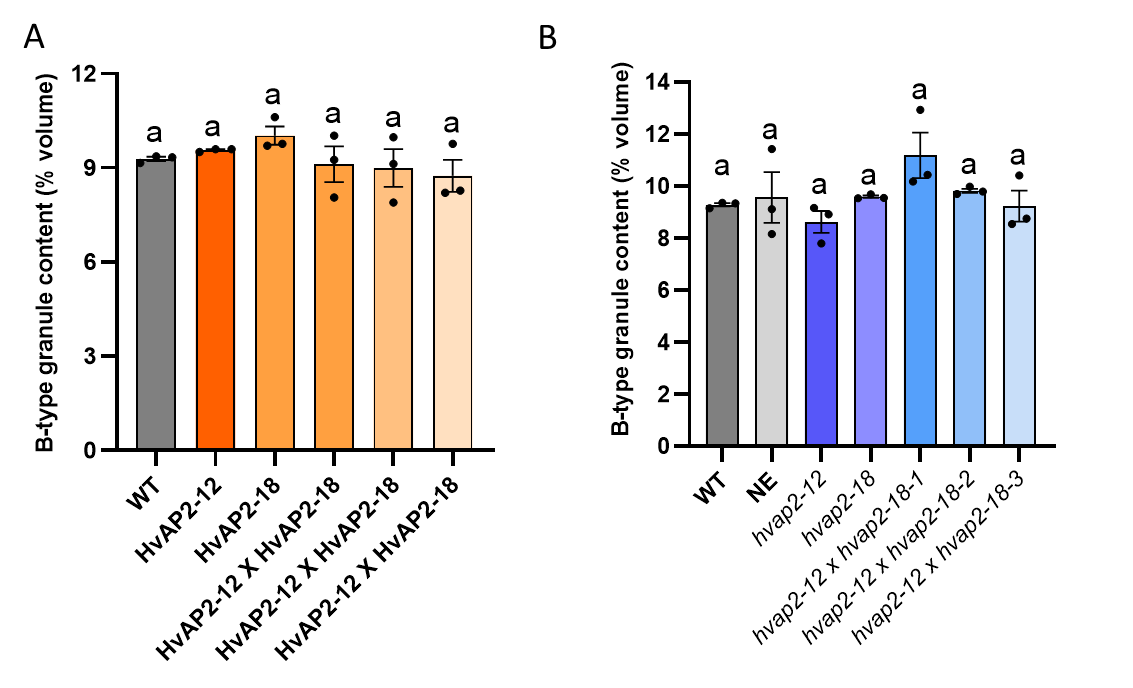


Figure S26. Analysis of starch granule content in double overexpression and mutant lines of *HvAP2-12* and *HvAP2-18*.
