# Supplementary material for: Dual‐Layer Transcriptional‐Protein Regulation by HvAP2‐12 Represses HvAP2‐18 Activity to Fine‐Tune Barley Starch Synthesis
Source: Plant Biotechnol J. 2025 Aug 23;23(12):5694–709. doi: 10.1111/pbi.70338 (PMC12665055; doi:10.1111/pbi.70338)
Supplement: Supplementary file 3 — Data S1: pbi70338‐sup‐0003‐Supinfo.docx. [file PBI-23-5694-s002.docx]

**Supplement Materials**

**Materials and methods**

**Plant materials**

Barley cv. ‘Golden Promise’ was used as the transgenic donor plant. The *HvAP2-12* and *HvAP2-18* coding regions were cloned to construct overexpression vectors, and the promoter utilized the 1500 bp upstream region of the target gene. Amplification primers are provided in Supplemental Table 1. The overexpression vector was pCAMBIA1302 (source). *HvAP2-12* and *HvAP2-18* gene-edited lines were developed using the vector VK005-05 (catalog no. VK005-05; Viewsolid Biotech, Beijing, China). Golden Promise plants and transgenic lines were cultivated in a Phytotron chamber under a photoperiod of 16 h light/8 h dark at temperatures of 24°C (day) and 18°C (night). Barley transformation was carried out as described by Hinchliffe and Harwood (2019). Further procedural details are described by Yang et al. (2022)(Yang et al., 2022).

*HvAP2-12* and *HvAP2-18* double-gene overexpression materials were generated. These materials underwent hybridization, followed by identification to obtain *HvAP2-12* and *HvAP2-18* double-overexpression lines. To create double-gene mutation lines, *HvAP2-12* and *HvAP2-18* single-deletion lines were hybridized, and F2 progeny were sequenced to identify double-gene mutants.

**Subcellular Localization**

For subcellular localization in *N. benthamiana* leaves, the full-length coding sequences of *HvAP2-12* and *HvAP2-18* were cloned into the pCAMBIA1302 vector, transformed into Agrobacterium tumefaciens strain GV3101, and infiltrated into the leaves at an optical density (OD600) of 0.6. For wheat protoplasts, *HvAP2-12* and *HvAP2-18* coding regions were cloned into pJIT163-GFP vectors and introduced into barley protoplasts via polyethylene glycol (PEG) mediation (Yoo et al., 2007). The GFP signal in protoplasts was visualized using laser confocal microscopy.

**Yeast One-Hybrid Screening and Assay**

Yeast one-hybrid screening was performed using the Matchmaker Gold Yeast One-Hybrid Library Screening System (Clontech, Palo Alto, CA). Fragments from the promoters of *HvAGP-S* and *HvSBE1* genes were subcloned into pAbAi vectors to generate decoy constructs. These constructs were linearized by digestion with *Bst*BI and integrated into the URA3-52 locus of the Y1H Gold yeast genome to produce Y1H decoy strains. The coding sequence of HvAP2-18 was cloned into the pGADT7 vector to generate the pGADT7-TF construct. These constructs or empty vectors were transformed into Y1H decoy strains and selected on synthetic dropout (SD) medium/-Ura-Leu plates containing 100 ng/mL Aureobasidin A (AbA).

**Phylogenetic Analysis**

To determine the evolutionary relationship between HvAP2-12 in barley and its homologous proteins in rice, maize, and wheat, homologous proteins of HvAP2-12 were downloaded from the NCBI database. ClustalW in MEGAX.8 software was used to align all amino acid sequences, and a phylogenetic tree was constructed using the neighbor-joining method based on the JTT matrix model with 1,000 bootstrap replicates.

**RT-qPCR**

Total RNA was extracted from 15 days post-anthesis (dpa) barley grain samples using the Plant RNA Kit (Biofit, Chengdu, China), and first-strand cDNA was synthesized using the HiScript II 1st Strand cDNA Synthesis Kit (Vazyme Biotech, R223-01) according to the manufacturer’s instructions. qRT-PCR analysis was conducted on the CFX 96 Real-Time System (Bio-Rad) using ChamQ Universal SYBR qPCR Master Mix (Vazyme Biotech, Q121-02/03). Data were analyzed using CFX Manager software (Bio-Rad) and the 2−∆∆Ct method. Three biological replicates were prepared for each transgenic line. Barley *β-actin* and *glyceraldehyde 3-phosphate dehydrogenase* (*GAPDH*) genes served as internal reference genes for normalizing the relative expression of candidate genes.

**Yeast Two-Hybrid Assay**

The *HvAP2-12* and *HvAP2-18* genes were cloned into the pGADT7-AD and pGBKT7-BD vectors, respectively. The constructs were transformed into the Y2H Gold strain and screened on SD/-Trp/-Leu medium following the manufacturer’s instructions (Clontech). Positive colonies with recombinant AD and BD vectors were grown on SD/-Trp/-Leu/-His/-Ade medium containing AbA and 20 mM X-α-gal to assess yeast growth and selective color reactions. The combinations pGADT7-T/pGBKT7-53 and pGADT7-T/pGBK-Lam were used as positive and negative controls, respectively.

**Dual Luciferase Transcriptional Activity Assay**

The pGreenII 62-SK and pGreenII 0800-LUC vectors were used for dual luciferase reporter assays. The open reading frames (ORFs) of *HvAP2-12* and *HvAP2-18* were cloned into the pGreenII 62-SK vector as effectors. Fragments corresponding to the 1,500 bp upstream regions of the starch synthase and hordein genes were amplified from Golden Promise genomic DNA and cloned into the pGreenII 0800-LUC vector as reporters. The effector and reporter plasmids were co-transformed into the epidermal cells of 4-week-old *N. benthamiana* plants mediated by A. tumefaciens strain GV3101. Firefly and Renilla luciferase activities were quantified using the Dual Luciferase Reporter Assay Kit (Vazyme Biotech) and a GloMax 96-microwell plate luminescence detector (Promega). The normalized data are presented as the ratio of firefly to Renilla luciferase signal intensity from three independent biological replicates.

**Transcriptome Deep Sequencing (RNA-seq)**

Total RNA was extracted from the developing grain of *HvAP2-12* and *HvAP2-18* overexpression lines, CRISPR mutant lines, and wild-type ‘Golden Promise’ at 15 dpa using the RNAprep Pure Plant Kit (Tiangen, Beijing, China). Sequencing libraries were prepared using the NEBNext Ultra RNA Library Prep Kit for Illumina (NEB, Ipswich, MA, USA) following the manufacturer’s recommendations. Clustering of the index-coded samples was performed using the cBot Cluster Generation System with the TruSeq PE Cluster Kit v4-cBot-HS (Illumina). The libraries were sequenced on an Illumina platform to generate paired-end reads. Clean reads were mapped to the reference genome sequence (PGSB_V2) using Hisat2 tools. Gene expression levels were quantified in fragments per kilobase of transcript per million fragments mapped (FPKM). Differential expression analysis between groups was performed using DESeq2, with a false discovery rate (FDR) < 0.01 and a fold change (FC) ≥ 2 as the significance threshold.

**Grain Phenotype Observation**

Grain length and width of the transgenic lines and wild-type plants were scanned using an Epson Perfection V700 Photo scanner (EPSON, Beijing, China) and analyzed using WinSEEDLE software. For each line, three biological replicates were analyzed.

**Determination of Starch**

Mature grains were ground into fine powder for starch determination. Total starch and amylose content were measured using the Total Starch Assay Kit and Amylose/Amylopectin Assay Kit (Megazyme, Wicklow, Ireland) following the manufacturer’s protocols.

**Scanning Electron Microscopy**

To purify starch from mature grains, grains (3 to 5 per extraction) were soaked overnight in ddH2O at 4 °C and homogenized using a mortar and pestle with excess ddH2O. Homogenates were filtered through a 70-µm nylon mesh, centrifuged at 3,000 × g for 5 min, and the starch pellet was resuspended in water. The suspension was centrifuged at 2,500 × g for 5 min on a 90% (v/v) Percoll cushion with 50 mM Tris-HCl (pH 8). The pellet was washed twice with 50 mM Tris-HCl (pH 6.8) containing 10 mM EDTA, 4% SDS (v/v), and 10 mM DTT, followed by two washes with ddH2O, and resuspended in ddH2O. Starch granule morphology was examined using a Nova NanoSEM 450 scanning electron microscope (FEI, Hillsboro). Granule size distributions were quantified using a Multisizer 4e Coulter counter (Beckman Coulter) with a 70-µm aperture tube. At least 100,000 particles were measured per sample. Relative volume versus diameter and relative number versus diameter plots were generated. A bimodal distribution (two log-normal distributions) was fitted to volume versus diameter plots to calculate mean diameters of A- and B-type granules and the B-type granule volume percentage using a Python script.

**Starch XRD diffraction analysis**

The crystalline morphology and degree of crystallinity of starch were analyzed using an X-ray diffractometer (Rigaku Corporation, Tokyo, Japan). Starch samples (approximately 100 mg) were ground, evenly spread, and tested. X-ray intensity was measured using Cu-Kα radiation (λ = 0.15406 nm) at a power of 1600 W (40 kV × 40 mA), with a scanning range of 4°–60° and a step size of 0.02°. The crystallinity and diffraction peaks were analyzed using MDI Jade 5.0 software.

**Analysis of Starch Chain Length Distribution**

Starch (10 mg) was dissolved in water by boiling for 60 minutes and treated with isoamylase (10 μL, 1400 U) in acetate buffer (0.6 M, pH 4.4). The debranched glucans were reduced with sodium borohydride and dried in vacuo. Samples were dissolved in 1 M NaOH, diluted, and analyzed using high-performance anion-exchange chromatography (HPAEC) on a CarboPac PA-200 column with a pulsed amperometric detector (Thermo Scientific, Dionex ICS 5000). Data were processed using Chromeleon 7.2 CDS.

**Statistical Analysis**

All experiments were conducted with at least three biological replicates. Data were presented as means ± standard deviation (SD). Differences between HvAP2-12, HvAP2-18, and wild-type lines were analyzed using Student’s t-test in SPSS v.20 (SPSS Inc., Chicago, USA), with significance set at P < 0.05.
